# Biological effects of carbon black nanoparticles are changed by surface coating with polycyclic aromatic hydrocarbons

**DOI:** 10.1186/s12989-017-0189-1

**Published:** 2017-03-21

**Authors:** Karina Lindner, Michael Ströbele, Sandra Schlick, Sina Webering, André Jenckel, Johannes Kopf, Olga Danov, Katherina Sewald, Christian Buj, Otto Creutzenberg, Thomas Tillmann, Gerhard Pohlmann, Heinrich Ernst, Christina Ziemann, Gereon Hüttmann, Holger Heine, Henning Bockhorn, Tanja Hansen, Peter König, Heinz Fehrenbach

**Affiliations:** 1 Institut für Anatomie, Zentrum für medizinische Struktur- und Zellbiologie, Universität zu Lübeck (UzL), Airway Research Center North (ARCN), German Center for Lung Research (DZL), Ratzeburger Allee 160, 23562 Lübeck, Germany; 2 Karlsruher Institut für Technologie, Engler-Bunte-Institut, Bereich Verbrennungstechnik, Karlsruhe, Germany; 3 Forschungszentrum Borstel, Leibniz-Zentrum für Medizin und Biowissenschaften, Experimentelle Pneumologie, Borstel, Airway Research Center North (ARCN), German Center for Lung Research (DZL), Borstel, Germany; 4 Forschungszentrum Borstel, Leibniz-Zentrum für Medizin und Biowissenschaften, Angeborene Immunität, Borstel, Airway Research Center North (ARCN), German Center for Lung Research (DZL), Borstel, Germany; 5 Fraunhofer Institut für Toxikologie und Experimentelle Medizin ITEM, Hannover, Biomedical Research in Endstage and Obstructive Lung Disease Hannover (BREATH), German Center for Lung Research (DZL), Hannover, Germany; 6 Institut für Biomedizinische Optik, Universität zu Lübeck (UzL), Lübeck, Airway Research Center North (ARCN), German Center for Lung Research (DZL), Lübeck, Germany

**Keywords:** Carbon black nanoparticles, Polycyclic aromatic hydrocarbons, Airway epithelial cells, Physicochemical characteristics, Mucociliary clearance, Cytotoxicity

## Abstract

**Background:**

Carbon black nanoparticles (CBNP) are mainly composed of carbon, with a small amount of other elements (including hydrogen and oxygen). The toxicity of CBNP has been attributed to their large surface area, and through adsorbing intrinsically toxic substances, such as polycyclic aromatic hydrocarbons (PAH). It is not clear whether a PAH surface coating changes the toxicological properties of CBNP by influencing their physicochemical properties, through the specific toxicity of the surface-bound PAH, or by a combination of both.

**Methods:**

Printex^®^90 (P90) was used as CBNP; the comparators were P90 coated with either benzo[a]pyrene (BaP) or 9-nitroanthracene (9NA), and soot from acetylene combustion that bears various PAHs on the surface (AS-PAH). Oxidative stress and *IL-8/KC* mRNA expression were determined in A549 and bronchial epithelial cells (16HBE14o-, Calu-3), mouse intrapulmonary airways and tracheal epithelial cells. Overall toxicity was tested in a rat inhalation study according to Organization for Economic Co-operation and Development (OECD) criteria. Effects on cytochrome monooxygenase (*Cyp*) mRNA expression, cell viability and mucociliary clearance were determined in acute exposure models using explanted murine trachea.

**Results:**

All particles had similar primary particle size, shape, hydrodynamic diameter and ζ-potential. All PAH-containing particles had a comparable specific surface area that was approximately one third that of P90. AS-PAH contained a mixture of PAH with expected higher toxicity than BaP or 9NA. PAH-coating reduced some effects of P90 such as *IL-8* mRNA expression and oxidative stress in A549 cells, granulocyte influx in the in vivo OECD experiment, and agglomeration of P90 and mucus release in the murine trachea ex vivo. Furthermore, P90-BaP decreased particle transport speed compared to P90 at 10 μg/ml. In contrast, PAH-coating induced *IL-8* mRNA expression in bronchial epithelial cell lines, and *Cyp* mRNA expression and apoptosis in tracheal epithelial cells. In line with the higher toxicity compared to P90-BaP and P90-9NA, AS-PAH had the strongest biological effects both ex vivo and in vivo*.*

**Conclusions:**

Our results demonstrate that the biological effect of CBNP is determined by a combination of specific surface area and surface-bound PAH, and varies in different target cells.

**Electronic supplementary material:**

The online version of this article (doi:10.1186/s12989-017-0189-1) contains supplementary material, which is available to authorized users.

## Background

The toxicity of nanoparticles is thought to be determined by their surface reactivity, influenced in turn by their chemical composition, physical properties and surface chemistry [1–8]. Carbon black nanoparticles (CBNP) are composed mainly of carbon with a small amount of other elements (including hydrogen and oxygen), and in themselves are nontoxic. However, CBNP are potentially harmful since their large surface area can interact with biological systems [1, 9–14]. Furthermore, substances such as polycyclic aromatic hydrocarbons (PAHs) that are intrinsically toxic can be adsorbed during CBNP synthesis [15]. The theory that the toxicity of CBNP can be increased by other substances has been validated by the application of metal ions, which increased the toxicity due to autophagy and lysosomal dysfunction [16]. However, it is still not clear how direct binding of chemical add-ons to CBNP modifies their interaction with biological systems [5, 17–19]. Several outcomes are theoretically possible. First, binding of chemicals to the particle surface can modify the surface properties of CBNP, reducing the surface area, thereby potentially reducing toxicological effects [1]. Second, the toxicity of the bound substances can determine the overall toxicity of the resulting particles [20]. Third, the modification could lead to a new particle type, whose biological activity might be a combination of changed physicochemical properties and the toxicity of the bound substances.

A common surface modification of CBNP is the adsorption of PAHs, occurring during incomplete combustion [21]. PAHs are thought to be mutagenic and carcinogenic, generally after toxification by cytochrome P450 enzymes (Cyp), and therefore are regarded as lung tumor inducing substances, although with only very limited acute toxicity [15, 22, 23]. Despite this limited acute toxicity, their effect on biological systems can be easily monitored by their potential to induce specific Cyp enzymes.

Compared with unmodified CBNP, surface modification of CBNP with PAHs has not shown increased tumor induction either following long-term exposure [24] or in cell lines in terms of cytokine release, induction of single and double strand breaks, cell cycle arrest, or phosphorylation of p53 or histone [25–27]. This suggests that PAHs have limited toxic effects when bound to CBNP, supporting the theory that they are not biologically available in this state [28] and that surface modification has a minor effect on the overall toxicity of CBNP.

However, diesel exhaust particles that contain PAHs are associated with an increased risk of asthma, and experiments involving exposure of a bronchial cell line to ultrafine particles implied a role of PAH in their toxic effects [29–31]. Furthermore, removal of PAHs from these exhaust particles resulted in reduced carcinogenic potential [24]. These data suggests that the effect of PAHs on the behavior of CBNP in biological systems is more complex.

Since inhalation is the predominant route of exposure to CBNP, we tested the toxicity of particles in different test systems including cell cultures, ex vivo studies on excised airways, and an in vivo inhalation study conducted according to Organization for Economic Co-operation and Development (OECD) criteria [32]. Initially, we tested the CBNP in high-throughput cell culture systems that can predict the toxicity of particles, and which therefore allowed us to preselect CBNP for in vivo testing. We then analyzed the in vivo effects of the selected CBNP in inhalation studies, and then finally tested the CBNP in ex vivo airway preparations to determine their impact on ciliary beat frequency, particle transport speed and mucus appearance. In every test system we analyzed parameters that indicate CBNP-induced oxidative stress and IL-8 response.

This allowed us to compare specific toxicological effects in each system to gain a more comprehensive insight into the cellular effects of the particles.

## Methods

To understand how surface modification can change the interaction of CBNP with biological systems we used PAHs to modify the surface of a toxicologically well-defined CBNP, Printex^®^90 (P90). This is characterized by a high surface area and has been widely used in toxicological studies, resulting in only minor toxic effects [1, 33, 34].

For modification of the P90 surface, we used benzo[a]pyrene (BaP) and 9-nitroanthracene (9NA). BaP was chosen because of the well characterized toxicity of its metabolites, which are known to induce ROS and DNA adducts [23, 35–38]. BaP is known to induce Cyp1A1 and 1B1, which then metabolize BaP to toxic metabolites; this therefore allows monitoring of BaP activity and its biological effect [39, 40]. In contrast, 9NA is a PAH that occurs during combustion, and is regarded as a low toxicity PAH, as predicted by the Ames test and human cell mutagenicity assay [41–43]. However, due to its nitro group other toxic mechanisms can occur induced by intermediates resulting from reduction of the nitro group [41]. As coating of a particle does not necessarily represent the situation found in nanoparticles that acquire PAH during synthesis, we also generated CBNP by acetylene combustion [44]. The resulting acetylene soot (AS) had a mixture of PAHs on the surface (AS-PAH). In the suspensions we used, AS-PAH had a slightly larger specific surface area, but similar aggregate size and ζ-potential compared to PAH-coated P90, The physicochemical parameters of the different particles were evaluated by a variety of analytic test methods (see Tables 1, 2 in the Result﻿s section﻿ and Additional files 1, 2).

## Particle synthesis and characterization

### Modification of CBNP

80 g of unmodified P90 (Evonik Carbon Black GmbH, Frankfurt, Germany) together with 20 g of BaP (Thermo Fisher Scientific-Alfa Aesar, Heysham, Lancashire, UK) or 9NA (Merck Schuchardt OHG, Hohenbrunn, Germany) were suspended in 100 ml dimethyl ether and incubated in an ultrasonic bath at room temperature for 15 min. After subsequent vacuum desiccation, the coated P90 nanoparticles were stored at room temperature.

### Gas phase synthesis of AS-PAH

A flat flame burner in a low pressure combustion chamber with acetylene as the fuel was used for the synthesis of AS-PAH. The experiments were carried out with a carbon to oxygen ratio of 1.1 to 1. The laminar mass flow of acetylene was set via mass flow controllers (Brooks 5850E; Brooks Instruments, Hatfield, PA, USA) on 88 l_n_/h with the oxygen mass flow adjusted to 80 l_n_/h. Temperature and pressure were monitored by a thermocouple and a pressure sensor, with the pressure set to 70 mbar during synthesis. The AS-PAH was collected for 60 min on a filter (Gore Membrane Filter Bags) between the combustion chamber and the rotary vane pump (TRIVAC D 65 B; Oerlikon Leybold Vakuum, Cologne, Germany).

### Particle size distribution

The particle size distributions were measured by scanning electron microscopy (SEM; Leo 1530; LEO Electron Microscopy Inc., Thornwood, NY, USA) and transmission electron microscopy (TEM; CM200 FEG/ST, Philips, Amsterdam, Netherlands). The PAHs on the particle surface had to be extracted with toluene prior to electron microscopy, as otherwise they would be cracked by the beam and the particles would grow. Remaining CBNP were then suspended with acetone in an ultrasonic bath, and the suspension was applied thinly using an ultrasonic nebulizer, either to a silicon wafer (for SEM) or to a 400 mesh cooper grid (Ted Pella, Inc., Redding, CA, USA) (for TEM). The diameter of 200 particles of 5 images was determined with the program ImageJ. A log normal curve was fitted on the particle size distribution to get the average particle diameter.

### Thermal analysis

The thermal release of coated PAHs from P90 and of volatile substances from AS-PAH was investigated using a thermogravimetric analyzer (DuPont 951; DuPont Company, Wilmington, DE, USA) connected to a quadrupole mass spectrometer (QMG 420; Balzers AG, Balzers, Liechtenstein) through a differentially pumped inlet system. The transfer line between the thermobalance and the mass spectrometer was heated up to 400 °C to prevent condensation of the pyrolysis products. The experiments were performed with sample sizes of 5–10 mg at a heating rate of 10 °C/min up to a final temperature of 800 °C at a helium flow rate of 100 ml/min. Each sample was measured at least three times.

### Identification of PAHs on AS-PAH surface

To characterize the PAHs on the particle surfaces, CBNP samples were heated under dynamic conditions. A sample of 100 mg was heated up in an oven in 10 °C/min increments to vaporize the PAHs, with the volatile PAHs collected in a cold trap. The condensed products were dissolved in 5 ml toluene, and were characterized by a standard gas chromatograph-mass spectrometer (GC/MS-QP 2010SE; Shimadzu Corporation, Tokyo, Japan). The chromatographic parameters for GC/MS were: Phenomenex zb-5msi column (column length: 30 m; I.D.: 0.25 mm; film thickness 0.25 mm); injector temperature 250 °C; oven temperature program: 65 °C at 5 min constant, 10 °C min to 350 °C at 10 min constant.

### Specific surface area

The surface area was determined by Brunauer, Emmett and Teller (BET) measurement using a BelSorp II device (BelJapan, Inc., Osaka, Japan), using the BelSorp analysis program.

### Particle size and ζ-potential in suspension

Suspensions were prepared with 10 mg of sample plus 100 ml of double-deionized water. To keep the suspension stable, 500 mg of bovine serum albumin (BSA) (AppliChemInc., Maryland Heights, MO, USA) was added. The suspensions were treated with an ultrasonic homogenizer (Sonoplus, Rod: VS 70 D; Bandelin electronic GmbH & Co. KG, Berlin, Germany) for 40 min in pulse mode, changing 20 times between 30 s ultrasonic and 90 s resting interval. To avoid thermal denaturation of BSA, the suspensions were cooled in a beaker containing ice water during treatment. The particle size measurements were carried out as soon as the suspension had reached room temperature using a Malvern ZetaSizer Nano-ZS (Malvern Instruments Ltd, Worcestershire, UK). Particle size was quantified by dynamic light scattering, and ζ-potential was measured by laser Doppler microelectrophoresis using the Smoluchowski model. The index of refraction and the absorption were preset at 2.0 in the measurement software (DTS Nano; Malvern Instruments Ltd, Worcestershire, UK). The cuvettes to measure the particle size distribution and the ζ-potential were washed using double-deionized water and ethanol (70%) to remove potential impurities. Subsequently, the cuvettes were rinsed using approximately 2 ml of the sample, and were then filled with the sample and placed in the Zetasizer Nano-ZS. The suspensions were characterized in triplicates, and the means and standard derivations were calculated.

### Endotoxin tests

CBNP suspensions were tested for endotoxins using the CROMO-LAL assay (Associates of Cape Cod Inc., East Falmouth, MA, USA) according to the manufacturer’s protocol. The limit of detection was 0.005 EU/ml (comparable to 0.5 pg/ml endotoxin of E. coli Type 055:B5). The suspensions were only used for experiments if no endotoxins were detected.

## Experiments with human pulmonary cell lines

### Cell culture

We used A549 cells (DSMZ, Braunschweig, Germany), 16HBE14o- cells (University California, San Francisco, USA; described in [45]) and Calu-3 cells (American Type Culture Collection, Manassas, VA, USA). The epithelial cells were cultured in Dulbecco’s modified eagle’s medium (DMEM) with 10% fetal calf serum (FCS) and 0.1% gentamicin for ROS and transepithelial electrical resistance (TEER) measurements. For mRNA expression analysis, A549 cells were cultured in DMEM (Fisher Scientific GmbH, Schwerte, Germany) with 10% FCS and 0.1% gentamicin, 16HBE14o- cells were cultured in DMEM (Biochrom AG, Berlin, Germany) with 10% FCS (PAA Laboratories GmbH, Cölbe, Germany) 1% L-glutamine (Gibco™, Life Technologies GmbH, Darmstadt, Germany) and 1% penicillin/streptomycin (Sigma Aldrich Chemie GmbH, Taufkirchen, Germany), and Calu-3 cells were cultured in RPMI 1640 containing stable glutamine supplemented with 10% FCS, 100 U/ml penicillin and 100 μg/ml streptomycin (all from Biochrom AG, Berlin, Germany). Cells were kept in a 37 °C humidified atmosphere containing 5% CO_2_.

### Flow cytometric measurements of reactive oxygen species (ROS)

Cells were seeded in T25 flasks (PAA Laboratories GmbH, Cölbe, Germany), at 0.6 × 10^6^ for A549 cells and at 1 × 10^6^ for 16HBE14o- cells. Subconfluent cultures were exposed to CBNP at a concentration of 10 μg/ml or 50 μg/ml for 24 h. The culture medium was subsequently removed and the cell monolayers were washed twice with 5 ml pre-warmed phosphate buffered saline (PBS). The fluorescent dye dichloro-dihydro-fluorescein diacetate (DCFH-DA) was applied to the cells in 5 ml fresh medium, with the resulting mixture incubated for 30 min at 37 °C. The culture medium was then removed and the cells were washed twice with 5 ml pre-warmed PBS and harvested by trypsinization. Flow cytometry measurements were done with a FACScan cytometer (Becton Dickinson Biosciences, San Jose, CA, USA) equipped with a 488 nm argon laser, with CELLQuest™ software used for data acquisition. For each sample, 10,000 events were analyzed and fluorescence emission of dichlorofluorescein (DCF) was examined at 530 nm.

### Quantitative RT-PCR

A549 cells were seeded in T25 flasks at 0.6 × 10^6^. Calu-3 cells and 16HBE14o- cells were seeded in 12-well plates (Costar, Corning, NY, USA) at 5 × 10^5^ cells and 1.6 × 10^5^ cells, respectively. After reaching a confluency of ≥90%, culture medium was exchanged and cells were treated with CBNP at 10 μg/ml, 50 μg/ml or medium/BSA alone for 24 h. Subsequently, cells were washed twice with pre-warmed medium or PBS to remove particles, and total RNA was extracted from Calu-3 cells using a NucleoSpin RNA Kit (Macherey-Nagel, Düren, Germany) and from A549 and 16HBE14o- cells using a RNeasy Kit (Quiagen GmbH, Hilden, Germany) according to the manufacturers’ instructions.

Reverse transcription was performed for RNA of A549 cells with the Omniscript Kit (Quiagen GmbH, Hilden, Germany), of 16HBE14o- cells using MaximaFirstStrand cDNA synthesis (Fermentas/Life Technologies GmbH, Darmstadt, Germany), and of Calu-3 cells using Oligo(dT)_12–18_ and Superscript III reverse transcriptase (Thermo Fisher Scientific, Waltham, USA), all according to the manufacturers’ protocols.

To quantify *IL-8* and hypoxanthine-guanine phosphoribosyltransferase (*HPRT*) mRNA levels in A549 cell preparations, we used Viia™7 (Life Technologies GmbH, Darmstadt, Germany); for preparations of 16HBE14o- and Calu-3 cells, we used LightCycler 480 SYBR green I Master (Roche Applied Science, Mannheim, Germany).

The sequences of used *IL-8* primers and housekeeping gene *HRPT1* primers are shown in Additional file 3. The housekeeping gene was evaluated with GenEx software (MultiD Analyses AB, Gothenburg, Sweden) [46]. The quality of amplificates was tested by melting curve analysis. The n-fold change was calculated for A549 cells with the program GenEx (GenEx Professional 5.4.3 Software), and by normalization of relative expression data to medium control data for 16HBE14o- and Calu-3 cells.

### Measurement of the transepithelial electrical resistance (TEER)

A total of 2 × 10^5^ Calu-3 cells were seeded per well on Transwell^®^ filter inserts (polyester, 12-well plates, 12 mm diameter, 0.4 μm pore size; Corning Costar, Bodenheim, Germany) and cultivated for 9 days. On day 10, the cell monolayers were exposed to CBNP at 10 μg/ml or 50 μg/ml for 24 h. The culture medium was changed and the cells were incubated for at least 15 min at 37 °C, before TEER was measured using an EVOM volt-ohm-meter (World Precision Instruments, Sarasota, Fl, USA).

### Statistical analysis

Differences between untreated controls and treated cultures were considered statistically significant at *p* < 0.05. Flow cytometric and TEER data were analyzed by two-sided Student’s t-test for unpaired values using Microsoft Excel. Relative mRNA expression results were analyzed by Mann Whitney U test.

## Nose-only inhalation study in rats

### Animals

Male Wistar rats (strain Crl:WI (Han)) were purchased from Charles River Deutschland (Sulzfeld, Germany). The age of the animals was 10 to 11 weeks and the weight 285 ± 24 g (mean ± SD) at the onset of exposure. Rats were exposed to the test item by nose-only inhalation. For 3 weeks prior to exposure, animals were trained to become accustomed to nose-only tubes. The OECD inhalation study on rats was approved by the Niedersächsisches Landesamt für Verbraucherschutz und Lebensmittelsicherheit (33.14 42502-04013/1199).

### Study design and dosing scheme

The CBNP were administered to the test animals by nose-only inhalation. P90, P90-BaP and AS-PAH were tested; P90-9NA was not administered, given the previously described low toxicity [41–43] and the in vitro tests in this study. A nominal aerosol concentration of 6 mg/m^3^ was used, with exposure of 6 h/day for two weeks (no exposure on weekends) according to the OECD criteria for acute exposure [32]. CBNP-related effects on innate immune cells in the bronchoalveolar lavage (BAL) and histopathological changes in lung tissue were analyzed on day 1 and day 14 after cessation of exposure. Potassium bromate (KBrO_3_) treated animals (250 mg/kg in physiological saline, intraperitoneal) served as positive controls for Comet-assay. The animals were treated with KBrO_3_ on day 1 and day 14. BAL was collected and analyzed 3 h after application.

### Bronchoalveolar lavage and leukocyte determination

BAL was performed in five rats per group after the end of exposure (day 1) and following a 14-day recovery period. Following preparation, lungs were lavaged with saline using two lavages of 5 ml each. The lavage fluid was collected in calibrated tubes and the harvested volume was recorded. Leukocyte concentration was determined using a counting chamber, and two cytoslides were prepared with a cytocentrifuge (Shandon Co., Frankfurt, Germany) for differential cell counts (macrophages, granulocytes, lymphocytes). The slides were air dried, and the cells were stained with Diffquick solutions (Medion Grifols Diagnostics AG, Düdingen, Switzerland) and counted. The results are presented as percentage of the total number of BAL cells.

### Statistical analysis

Differences between BAL analysis groups were considered statistically significant at *p* < 0.05. Data were analyzed using analysis of variance. If the group means differed significantly by the analysis of variance the means of the treated groups were compared with the means of the control groups using Dunnett’s test.

## Experiments with explanted mouse trachea

### Particle suspension in cell culture medium

For the ex vivo studies we used CBNP concentrations of 10 and 30 μg/ml. The concentration of 30 μg/ml was calculated by surface correction of the in vitro concentration.

The CBNP suspensions were diluted in DMEM (Gibco™, Life Technologies GmbH, Darmstadt, Germany) with 20 mM HEPES, 1% penicillin/streptomycin (Sigma Aldrich Chemie GmbH, Taufkirchen, Germany) and 2 mM L-glutamine (Life Technologies GmbH, Darmstadt, Germany) for incubation of tracheae.

### Animals

We used 8 to 12 week old female Balb/c mice (Charles River Laboratories, Sulzfeld, Germany). Mice were euthanized with an overdose of isoflurane for explantation of the trachea. The ex vivo studies on mouse tracheae were approved by the Ministerium für Landwirtschaft, Umwelt und ländliche Räume des Landes Schleswig-Holstein.

### Preparation and culture conditions of explanted trachea

Tracheae were explanted and transferred to culture dishes coated with a thin layer of Sylgard polymer and filled with medium. Each trachea sample was fixed on the bottom of a well with an insect needle, covered with 1 ml of medium with or without suspended CBNP and incubated at 37 °C for 24 h.

### Qualitative RT-PCR

To examine differences in mRNA expression levels, tracheae were cut into two pieces. One piece was incubated for 24 h with particle suspension; the other piece was incubated in medium only, serving as negative control. The epithelium was then removed using a sterile swab (Collection Swab H268A; Copan diagnostic, Inc., Murrieta, CA, USA). This isolation method was previously described in [47]. The RNA was isolated with innuPREP RNA isolation Kit (Analytic Jena AG, Jena, Germany) according to the manufacturer’s protocols. Any remaining DNA was enzymatically degraded using Amplification Grade DNase I kit AMPD-1 (Sigma-Aldrich Chemie GmbH, Steinheim, Germany). The quality and quantity of isolated mRNA was analyzed with a NanoDrop spectrophotometer (Thermo Fisher Scientific, Waltham, MA, USA). The mRNA was subsequently transcribed to cDNA using RevertAid H Minus Reverse Transcriptase (Fisher Scientific-Germany GmbH, Schwerte, Germany) mixed in transcription buffer with dNTPs (0.6 mM) (Fisher Scientific-Germany GmbH, Schwerte, Germany), DTT (10 mM) (Life technologies GmbH, Darmstadt, Germany) and random hexamer primers (17.5 ng/μl) (Biomers.net GmbH, Ulm, Germany).

Real time RT-PCR was performed in TaqMan Universal PCR Master Mix (Life technologies GmbH, Darmstadt, Germany), with probes tagged with reporter 6-FAM and quencher Tamra. The TaqMan probes and primers used for quantitative real time RT-PCR are shown in Additional file 3. The PCR program was: one cycle at 95 °C for 10 min followed by 50 cycles at 95 °C for 45 s and at 60 °C for 1 min. Changes in the transcript levels were determined by calculating the difference in cycle threshold to the respective negative control with the ΔΔC_t_ method and represented as n-fold expression (2 ^−ΔΔCt^).

### Apoptosis and necrosis assays

Tracheae were explanted, incubated in pre-warmed medium, opened and cut in four equal parts. One part was cultured in medium alone, two parts were incubated for 24 h with CBNP suspensions of 10 μg/ml and 30 μg/ml, respectively, and the remaining parts were incubated for 24 h with 1 μM staurosporine (Roche Diagnostics GmbH, Mannheim, Germany) or 1 mM paraquat (Sigma-Aldrich GmbH, Seelze, Germany), respectively as positive controls for the staining procedure.

For the apoptosis analysis, whole mount samples of tracheae were fixed with phosphate buffered 4% paraformaldehyde (pH 7.4), followed by washing with PBS (pH 7.4) and permeabilization with ice cold acetone. The specimens were then incubated overnight at room temperature with rabbit anti-cleaved caspase-3 antibody (#9661, dilution 1:300, Cell Signaling Technology Inc., Danvers, MA, USA). The primary antibody was detected using Alexa555-conjugated donkey anti-rabbit IgG (dilution 1:800, Life Technologies GmbH, Darmstadt, Germany).

For membrane damage analysis, ethidium homodimer-1 (1 μg/ml, Life Technologies GmbH, Darmstadt, Germany) in medium was added to the trachea preparation, and was then incubated for 30 min at 37 °C. The samples were fixed with 4% paraformaldehyde (pH 7.4), washed with PBS (pH 7.4) and permeabilized with acetone.

To identify the epithelial layer, ciliated cells were labeled with a mouse monoclonal antibody against acetylated α-tubulin (Clone 6-11B-1, Sigma-Aldrich GmbH, Steinheim, Germany) that was directly labeled with Atto488 (Lightning-Link™Atto488 conjugation kit; Innova Bioscience Ltd., Cambridge, UK); cell nuclei were visualized using Hoechst 33258 (0.1 μg/ml; Sigma-Aldrich, Steinheim, Germany) (Additional file 4).

Apoptosis and cell membrane damage were analyzed using a confocal laser scanning microscope (LSM 510 Meta; Carl Zeiss MicroImaging GmbH, Göttingen, Germany) equipped with a Plan-Apochromat 20×/0.75 or C-Apochromat 40×/1.2 W objective. The total number of apoptotic and necrotic epithelial cells was counted in the complete whole mount preparation.

### Ciliary beat frequency and cilia-driven particle transport

Tracheae were transferred to a Delta T4 Culture dish (Bioptechs Inc., Butler, PA, USA), the bottom of which was covered with sylgard polymer. Samples were fixed with insect needles with the epithelium facing upwards, and were then covered with 2 ml of HEPES-Ringer solution. The temperature of HEPES-Ringer solution was maintained at 30 °C using a Bioptechs Delta T4 Culture Dish Controller (Bioptechs Inc., Butler, PA, USA), and the epithelial surface was imaged using a Zeiss Axioskop 2 FS fixed stage microscope equipped with Achroplan 40×/0.80 W or 20×/0.50 W objectives (Carl Zeiss MicroImaging GmbH, Göttingen, Germany).

Polystyrene particles (4.5 μm) were added to the HEPES-Ringer solution with a concentration of 2 × 10^6^ particles/ml. The transport of particles was recorded with a SMX-150 M camera (EHD Imaging GmbH, Damme, Germany). Movies (each with 200 images) of polystyrene particle transport were recorded from at least six different areas for each trachea at 12 Hz frame rate and a resolution of 1280 × 1024 pixels. Particle transport speed was determined by tracking individual particles and manually checking each recorded track using Image-Pro^®^ Plus 6.0 (Medium Cybernetics, Inc., Bethesda, MD, USA). The mean particle transport speed was calculated for each experiment. Movies (each with 1000 images) of cilia motion were recorded at 100 Hz frame rate and 640 × 480 pixels). The ciliary beat frequency was determined by Fourier transformation of grey level changes over time using an in-house developed software written in Mathlab (Mathworks, Natick, MA, USA). For visualization of ciliary beat frequency, the dominant frequency, i.e. the frequency with the highest amplitude, was identified for every pixel in the movie and color-coded. Movies were recorded from at least six different areas for each trachea. The ciliary beat frequency of a minimum of 10 ciliated cells was measured in every movie and the mean frequency from these cells was calculated. To increase ciliary beat frequency or cilia-driven particle transport, ATP (10 μM) was applied as positive control.

### Statistical analysis

All ex vivo experiments were carried out at least three times. Quantitative RT-PCR data are presented as mean ± SEM. The data sets were analyzed using paired-sample Wilcoxon signed-rank test in GraphPad Prism 5 (GraphPad Software, Inc., La Jolla, CA, USA). *P* values < 0.05 were considered statistically significant.

### Mucus staining in tracheal whole mounts

Tracheae were incubated in 1 ml HEPES-Ringer solution at 30 °C; 1 μl of a 1:1 mix of wheat germ agglutinin (WGA) and Ulex europaeus agglutinin (UEA-1) was added (each 1 μg/ml). WGA stains N-acetyl-D-glucosamine and sialic acid residues, whereas UEA-1 has an affinity for L-fucose, all of which are carbohydrate residues present in mucins [48, 49]. In addition, WGA labels ciliated cells [50]. The staining was imaged with an AxioMR camera and an Achroplan 40×/0.8 W objective with a resolution of 1388 × 1040 pixels.

Further information on the determination of cytokine levels and cell viability in vitro, CBNP aerosol generation and exposure of rats, oxidative Comet-Assay, histopathology of the rat lungs, particle suspensions in cell culture medium, preparation and culture conditions of intrapulmonary airways, qualitative RT-PCR, macroscopic analysis, scanning electron microscopy and individual statistics are provided in Additional file 5.

## Results

### Characterization of CBNP and their suspensions

All of the tested nanoparticles had similar hydrodynamic diameters and ζ-potentials in medium (Table 1 and Additional file 1). P90 without PAHs had a mean primary particle size of 16.5 nm (Table 1 and Additional file 1). The minimal mass loss at the final temperature of 800 °C indicated that P90 had negligible volatile compounds on the surface. The coating of P90 nanoparticles with BaP or 9NA reduced the specific surface area about one third. P90-BaP and P90-9NA had almost the same mass loss, indicating a similar PAH proportion on the CBNP surface (Table 1 and Additional file 2A).

**Table 1:** Characteristics of dry and suspended CBNP

Particle properties	P90	P90-BaP	P90-9NA	AS-PAH
Mean primary particle size (nm)	16.5 ± 0.4^a^	16.5 ± 0.4^a^	16.5 ± 0.4^a^	14.2 ± 0.1^a^
Mass loss (%)	0.5 ± 0.4	16.1 ± 0.5	14.6 ± 0.1	19.0 ± 0.7
Specific surface area (m^2^/g)	302 ± 16	91 ± 2	91^#^	115 ± 3
Hydrodynamic diameter in water/BSA (nm)	155 ± 1	176 ± 5	172 ± 7	174 ± 5
ζ-potential in water/BSA (mV)	−34 ± 5	−38 ± 4	−37 ± 3	−36 ± 3
Hydrodynamic diameter in medium (nm)	163 ± 6	160 ± 8	156 ± 11	170 ± 4
ζ-potential in medium (mV)	−15 ± 1	−13 ± 1	−13 ± 1	−12 ± 1

Data are mean ± SEM. ^a^measurement without PAHs; *n* = 3–13, except ^#^ *n* = 1

AS-PAH exhibited a slightly larger specific surface area and mass loss compared with the coated P90 nanoparticles, and AS-PAH without PAHs showed a slightly smaller mean primary particle size than uncoated P90 (Table 1 and Additional file 1).

The thermogravimetric (TG) analysis of AS-PAH indicated a two-stage thermal desorption of surface components (Additional file 2B). The first stage ended at approximately 240 °C and mainly included PAHs (Additional file 2C). Because of possible defragmentation, the exact PAH content cannot be determined, but a minimal PAH content of approximately 11% was calculated via TG curve correlating with the mass loss at 240 °C (Additional file 2C). In the second stage, hydrogen, methane, water, acetylene and carbon dioxide were mainly released (Additional file 2D).

With combined TG/MS analysis, we detected PAHs with a maximal mass-to-charge ratio of 202 (Additional file 2E). Due to the possible condensation of high weight PAHs with a boiling point with more than 400 °C before MS inlet, we extracted the PAHs from AS-PAH and analyzed them with gas chromatographic (GC)/MS. The results are reported in Table 2 and Additional file 2 F. Endotoxins were not detected in any CBNP test preparation.

**Table 2:** Identified PAHs extracted from the AS-PAH surface

Peak	m/z	Retention time (min)	Name	Sum formula	Rings	IARC carcinogens classification	Identified by
1	128	10.867	Naphthalene	C_10_H_8_	2	2B	M
2	152	14.250	Biphenylene	C_12_H_8_	3	–	NIST
3	152	14.900	Acenaphthylene	C_12_H_8_	3	–	M
4	166	16.583	Fluorene	C_13_H_10_	3	3	M
5	178	18.867	Phenanthrene	C_14_H_10_	3	3	NIST
6	178	18.958	Anthracene	C_14_H_10_	3	3	M
7	190	20.308	Benzo[def]fluorene	C_15_H_10_	4	–	NIST
8	202	21.725	Fluoranthene	C_16_H_10_	3	3	M
9	202	22.233	Pyrene	C_16_H_10_	4	3	M
10–12	226	24.667–25.125	Cyclopenta[cd]pyrene/Benzo[ghi]fluoranthene	C_18_H_10_	5	2A 3	NIST
13	240	26.450	9H-Cyclopenta[a]pyrene	C_19_H_12_	5	–	NIST
14	252	28.225	Benzo[j]fluoranthene	C_20_H_12_	5	2B	NIST
15	276	30.775	Benzo[ghi]perylene	C_20_H_12_	6	3	NIST
16	276	30.992	Indeno[1,2,3-cd]pyrene	C_20_H_12_	6	2B	NIST
17	300	33.150	Coronene	C_24_H_12_	7	3	NIST

PAH compounds were analyzed with GC/MS and identified by reference substances (M) or by the National Institute of Standards and Technology Database (NIST). Some of the compounds are represented as isomers. A representative GC/MS chromatogram of PAHs from an AS-PAH sample is shown in Additional file 2 F. PAHs are classified by International Agency for Research on Cancer (IARC) monographs: 2A means “probably carcinogenic to humans”; 2B means “possibly carcinogenic to humans” and 3 means “not classifiable as to its carcinogenicity to humans” [90]. m/z = mass-to-charge ratio

## Analysis of CBNP effects in vitro

### The in vitro effects of CBNP depended on the cell line

A549 cells are widely accepted as a general model for lung epithelium. ROS formation was increased in these cells by P90 and AS-PAH, but not by P90-BaP or P90-9NA (Table 3). The highest ROS levels were detected after treatment with AS-PAH at both dose levels (Table 3), although P90 was the only particle that induced mRNA expression of *IL-8* (Table 3).

**Table 3:** In vitro results of ROS, *IL-8* mRNA expression and TEER measurement after exposure to CBNP

		A549 cells		16HBE14o- cells		Calu-3 cells	
		Relative DCF fluorescence	*IL-8*	Relative DCF fluorescence	*IL-8*	TEER (%)	*IL-8*
P90	10 μg/ml	1.2 ± 0.2	1.2 ± 0.4	1.5 ± 0.2**	1.4 ± 0.2	103 ± 7	1.0 ± 0.2
	50 μg/ml	2.1 ± 0.5*	2.1 ± 0.8**	3.2 ± 1.1**	1.7 ± 0.9	109 ± 8	1.1 ± 0.0
P90-BaP	10 μg/ml	1.1 ± 0.1	0.7 ± 0.2	1.3 ± 0.1	0.7 ± 0.3	97 ± 8	1.9 ± 0.6**
	50 μg/ml	1.2 ± 0.1	0.7 ± 0.2	2.1 ± 0.1**	1.9 ± 1.8	94 ± 9	3.0 ± 0.9**
P90-9NA	10 μg/ml	0.9 ± 0.1	0.8 ± 0.2	1.1 ± 0.1	1.1 ± 0.5	105 ± 10	1.1 ± 0.1
	50 μg/ml	1.2 ± 0.2	0.8 ± 0.2	2.1 ± 0.3**	2.0 ± 0.9*	104 ± 9	1.2 ± 0.1
AS-PAH	10 μg/ml	2.3 ± 0.2**	0.8 ± 0.3	1.8 ± 0.2**	1.1 ± 0.4	91 ± 7*	1.1 ± 0.2
	50 μg/ml	3.0 ± 0.4**	0.8 ± 0.3	1.9 ± 0.1**	1.4 ± 0.8	90 ± 6*	2.1 ± 1.2**

Data show the n-fold change compared to medium controls of relative DCF fluorescence in A549 and 16HBE14o- cells, the n-fold change of relative *IL-8* mRNA expression in A549, 16HBE14o- and Calu-3 cells, respectively, and the changes of TEER in Calu-3 cells after 24 h CBNP exposure

Data are mean ± SD. *n* = 3–5, *p < 0.05 and **p < 0.01 CBNP compared to medium controls analyzed by two-sided Student’s t-test for unpaired values (ROS analysis and TEER results) or Mann Whitney U test (mRNA expression data)

16HBE14o- cells are regarded as model for bronchial epithelial cells. In these cells, all CBNP induced ROS formation at 50 μg/ml, but only P90-9NA increased *IL-8* mRNA expression. Calu-3 cells are models for both bronchial epithelial cells and epithelial barrier function. In these cells, P90-BaP and AS-PAH induced *IL-8* mRNA expression; only AS-PAH reduced TEER (Table 3). AS-PAH-induced *IL-8* protein release was significantly increased compared to that following P90 exposure (Additional file 6A). *IL-6* protein was significantly increased in the supernatant after exposure to 50 μg/ml P90-BaP, and following exposure to both concentrations of AS-PAH (Additional file 6B). Cell viability was not significantly affected by the tested CBNP at either concentration (Additional file 6C).

## In vivo nose-only inhalation study in rats

### CBNP aerosol characterization

Aerosol concentrations and mass median aerodynamic diameters (MMADs) are summarized in Additional file 7. The mean concentrations were close to the target concentration (6 mg/m^3^) for all aerosols, and all MMADs were below 3 μm.

### Only AS-PAH increased the relative lung weights and induced a moderate inflammatory response

**Fig. 1:**
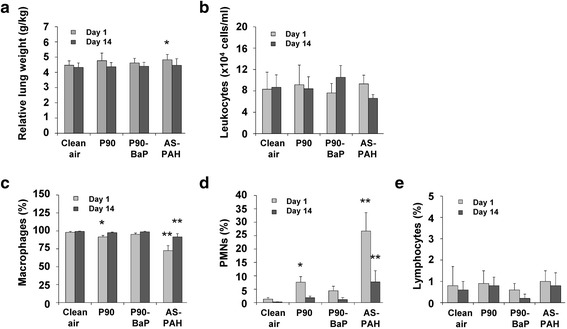
P90 and AS-PAH induced an inflammatory response in vivo. The diagrams show relative lung weights (**a**), the amount of total cells (**b**) and results of differential cell counts in BAL fluid (**c**-**e**) after nose-only inhalation. Data are mean ± SD; *n* = 10 results of relative lung weight; *n* = 5 for total cell count and differential cell counts; *p < 0.05 and **p < 0.01 CBNP exposure compared to clean air control, analyzed by analysis of variance and Dunnett’s test; PMNs = polymorphonuclear cells, D1 = day 1 post-exposure, D14 = day 14 post-exposure

On day 1 after cessation of exposure, relative lung weights were significantly increased only in the AS-PAH group (Fig. 1), with absolute lung wet weights increased in both the AS-PAH and P90-BaP inhalation group (Additional file 8). After a 14 day recovery period, the only effect seen in the inhalation groups was a slight decrease in the terminal body weight in the AS-PAH group (Fig. 1).

On day 1 post-exposure, there was a statistically significant increase in polymorphonuclear granulocyte numbers in the animals exposed to P90 and AS-PAH compared to the clean air control group (Fig. 1). In AS-PAH treated animals, numbers of polymorphonuclear granulocytes were still moderately increased on day 14 compared to clean air treated animals, indicating the presence of a mild chronic inflammatory response (Fig. 1). None of the tested CBNP influenced LDH, β-glucuronidase activity, or total protein levels in BAL fluid (Additional file 9A), and none induced oxidative DNA damage in BAL cells (Additional file 9B).

CBNP exposure induced very slight histological alterations (Additional file 10). However, only AS-PAH induced bronchiolo-alveolar hyperplasia, observed in two of five animals on day 14 post-exposure (Additional file 10).

### Ex vivo airway models

As the in vivo experiments did not specifically address airway epithelial function but in vitro experiments indicated that airway epithelial cells are sensitive to CBNP effects, we examined the particle effects on explanted murine airways.

### Only AS-PAH caused a pro-inflammatory response in the tracheal epithelium

Compared to medium controls, only AS-PAH significantly increased the mRNA expression of pro-inflammatory *IL-6* (30 μg/ml), and of keratinocyte chemoattractant (*KC*), the murine homologue of human IL-8) (Table 4). In contrast, P90-BaP and P90-9NA decreased *IL-6* mRNA expression at 30 μg/ml (Table 4). No increase of *KC* or *IL-6* mRNA was detected in the intrapulmonary airways, indicating that the effects observed are region specific (Additional file 11).

**Table 4:** Results of mRNA expression in tracheal epithelial cells after CBNP exposure

	P90		P90-BaP		P90-9NA		AS-PAH	
	10 μg/ml	30 μg/ml	10 μg/ml	30 μg/ml	10 μg/ml	30 μg/ml	10 μg/ml	30 μg/ml
*KC*	1.1 ± 0.2	1.1 ± 0.2	1.2 ± 0.2	0.7 ± 0.2	1.3 ± 0.2	1.2 ± 0.3	3.8 ± 1.5*	2.4 ± 0.6*
*IL-6*	0.7 ± 0.2	0.8 ± 0.1	1.0 ± 0.2	0.6 ± 0.1*	1.3 ± 0.3	0.7 ± 0.1*	2.0 ± 0.8	2.6 ± 0.4*
*Gpx3*	2.2 ± 0.9	0.7 ± 0.1*	0.8 ± 0.2	0.8 ± 0.1	1.7 ± 0.7	0.7 ± 0.1	5.2 ± 3.8	1.3 ± 0.5
*Gr*	0.7 ± 0.1*	0.7 ± 0.1*	1.0 ± 0.1	1.0 ± 0.1	1.4 ± 0.2	1.0 ± 0.2	1.5 ± 0.3	1.7 ± 0.5
*Cyp1a1*	1.4 ± 0.4	0.8 ± 0.3	331 ± 22*	1289 ± 412*	2.0 ± 1.0	30 ± 9*	1157 ± 232*	2442 ± 452*
*Cyp1b1*	0.2 ± 0.1*	0.2 ± 0.0*	5.3 ± 1.0*	10.9 ± 2.4*	1.3 ± 0.1	2.5 ± 0.6*	11.1 ± 4.3*	4.3 ± 0.7*

Data show the n-fold mRNA expression after CBNP exposure compared to medium controls. Exposure time was 24 h. Data are mean ± SEM. *n* = 5–7, *p < 0.05 CBNP compared to medium controls analyzed by Wilcoxon signed-rank test

*Gpx3* Glutathione peroxidase 3, *Gr* Glutathione reductase, *KC* keratinocyte chemoattractant, *IL-6* interleukine-6, *Cyp1a1*/*Cyp1b1* Cytochrome P450 subtypes 1a1 and 1b1

### All PAH containing CBNP induced *Cyp*-expression but none of the CBNP induced mRNA for indicators of oxidative stress in tracheal epithelial cells

We analyzed the mRNA expression of the cytochrome P450 enzymes Cyp1A1 and Cyp1B1 as indicators of PAH metabolism, and of Gpx3 and Gr as indicators of oxidative stress.

All PAH-containing CBNP induced *Cyp1a1* and *Cyp1b1* mRNA in the tracheal epithelium (Table 4). The most potent CBNP was AS-PAH, followed by P90-BaP. P90-9NA only induced *Cyp1a1* and *Cyp1b1* mRNA expression at 30 μg/ml. In contrast, the uncoated P90 nanoparticles did not induce *Cyp1a1* mRNA expression, and significantly decreased *Cyp1b1* mRNA expression compared to medium controls in tracheal epithelial cells (Table 4). None of the CBNP increased the mRNA expression of *Gpx3* or *Gr*. In intrapulmonary airways PAH-containing CBNP induced a similar expression pattern of *Cyp1a1* mRNA compared to the tracheal epithelium; no increase of mRNAs for anti-oxidative enzymes was detected (Additional file 11).

Since the mRNA data of *Cyp*-expression indicated that the CBNP affect airway epithelial cells and to detect changes in epithelial function, we assessed their impact on cilia-driven transport.

### Only P90 forms microscopically visible agglomerates; these bind to mucus and interact with ciliated cells

**Fig. 2:**
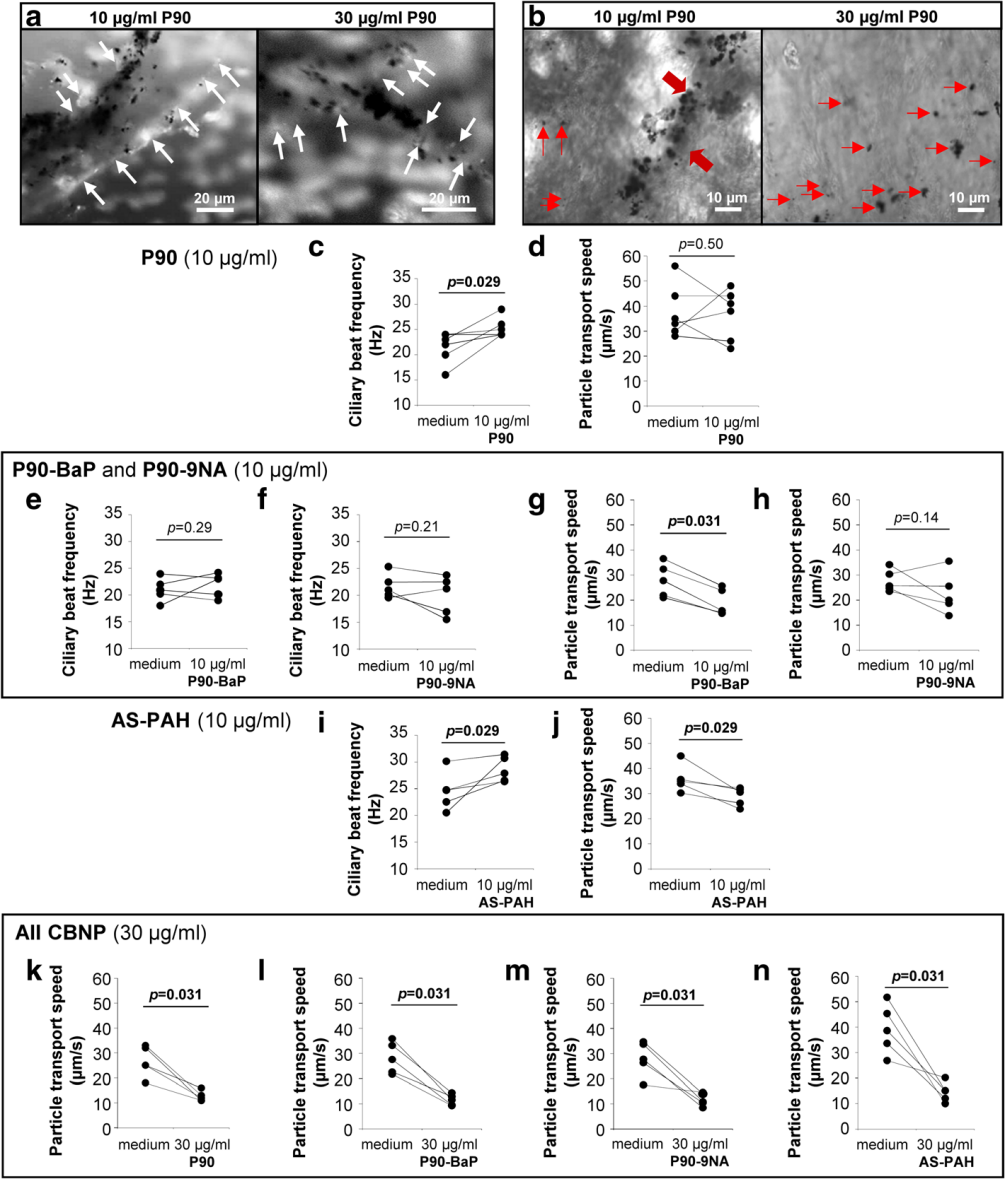
PAH altered P90 induced effects on mucociliary clearance in the ex vivo murine trachea. **a** Mucus on the epithelium was stained with a mixture of wheat germ agglutinin (WGA) and Ulex europaeus agglutinin-1 (UEA-1) after exposure to P90. White arrows indicate mucus structures associated with P90 agglomerates. **b** P90 agglomerates attached to cilia of ciliated epithelial cells. Red arrows indicate P90 agglomerates. **c**, **d** Ciliary beat frequency (**c**) and particle transport speed (**d**) after exposure to 10 μg/ml P90. **e**-**h** Ciliary beat frequency (**e**, **f**) and particle transport speed (**g**, **h**) after exposure to 10 μg/ml P90-BaP or P90-9NA. **i**, **j** Ciliary beat frequency (**i**) and particle transport speed (**j**) after exposure to 10 μg/ml AS-PAH. **k**-**n** Particle transport speed after exposure to 30 μg/ml P90 (**k**), P90-BaP (**l**), P90-9NA (**m**) and AS-PAH (**n**). **c**, **e**, **f**, **i** Each point represents the mean ciliary beat frequency of at least 50 ciliated cells measured at eight different tracheal regions of each animal. **d**, **g**, **h**, **k**-**n** Each point represents the mean particle transport speed of added polystyrene particles measured at eight different tracheal regions of each mouse. A minimum of *n* = 5 animals were analyzed. The exposure time was 24 h for all experiments. *p* < 0.05 was considered statistically significant. The medium controls were compared to CBNP exposure analyzed by Wilcoxon signed-rank test



On microscopic examination of tracheae, visible agglomerates were observed on the airway epithelium after 24 h of incubation with P90 at 10 μg/ml and 30 μg/ml (Additional file 12A). These agglomerates were bound either to mucus on the epithelium or directly to cilia of ciliated cells (Fig. 2a and b and Additional files 13 and 14). None of the PAH-containing CBNP showed microscopically visible agglomerates at either tested concentration (Additional file 12A).

**Additional file 13:** P90 agglomerates attached to cilia of ciliated cells after incubation with 10 μg/mlP90. The movie shows cilia movement and attached P90 agglomerates on cilia of tracheal epithelium after 24 h exposure to 10 μg/ml P90. The movie was recorded with 100 Hz and a resolution of 640 × 480 pixels. The image sequence contains 1000 images. (WMV 11853 kb)




**Additional file 14:** P90 agglomerates attached to cilia of ciliated cells after incubation with 30 μg/ml P90. The movie shows the cilia movement and bound P90 agglomerates on the cilia of tracheal epithelium after 24 h exposure to 30 μg/ml P90. The movie was recorded with 100 Hz, a resolution of 640 × 480 pixels and contains 1000 images. (WMV 11658 kb)


### At 10 μg/ml, P90 agglomerates increased ciliary beat frequency but mucus reduced particle transport speed

Compared to medium control, P90 at 10 μg/ml increased mean ciliary beat frequency (Fig. 2c), although it did not increase the mean of cilia-driven particle transport speed (Fig. 2d). Cells with bound agglomerates exhibited a higher ciliary beat frequency compared to cells without visible agglomerates (Additional file 12B and C). Furthermore, particle transport speed was reduced in areas with mucus and increased in areas without mucus compared to medium control (Additional file 12D). Incubation with P90 at a concentration of 1 μg/ml did not result in formation of agglomerates, and therefore did not increase ciliary beat frequency (Additional files 12A and 15A). However, this concentration was sufficient to increase mucus (Additional files 15B and C), and consequently to reduce particle transport speed (Additional file 15D).

### At 10 μg/ml, PAH-coated P90 did not increase ciliary beat frequency but differently influenced mucus release and particle transport speed

As expected from the inability to form agglomerates, neither P90-BaP nor P90-9NA increased ciliary beat frequency (Fig. 2e and f). Incubation with P90-BaP increased amounts of mucus on the epithelial cells (Additional files 16 and 17), with a consequent reduced particle transport speed compared to control (Fig. 2g and h). In contrast to P90 at 10 μg/ml, we observed dead cells on the epithelium during the experiments with both coated particles (Additional files 16, 17 and 18).

### At 10 μg/ml, AS-PAH increased ciliary beat frequency and mucus release but reduced particle transport speed

Although AS-PAH did not form agglomerates, it increased ciliary beat frequency and resulted in mucus release (Fig. 2i, and Additional files 12A and 19). Compared to control, AS-PAH reduced particle transport speed (Fig. 2j), and, as with both coated particles, we observed dead cells on the epithelium (Additional file 19).

### At 30 μg/ml, all particles resulted in reduced particle transport speed

At 30 μg/ml, none of the particles increased ciliary beat frequency but all reduced particle transport speed (Fig. 2k to n and Additional file 20). All particles increased mucus, and dead cells were visible on the epithelium (Additional files 16, 17, 18 and 19).

### PAH-containing particles induced apoptosis in tracheal epithelial cells

**Fig. 3:**
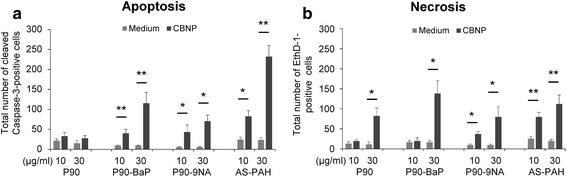
Surface PAHs increased epithelial toxicity of CBNP in the ex vivo murine tracheal model. The diagrams show the quantification of apoptotic (**a**) and necrotic (**b**) epithelial cell numbers. Data represent the total number of necrotic and apoptotic cells counted in whole mount preparation after 24 h exposure to CBNP compared to medium controls. All data are mean ± SEM. *n* = 5–11, *p < 0.05 and **p < 0.01, CBNP compared to medium controls analyzed by Wilcoxon signed-rank test

At 10 μg/ml, P90 did not increase cell death (Fig. 3a and b). In contrast, at this concentration all PAH-containing particles increased cell death, with inducing apoptosis (Fig. 3a) and P90-9NA and AS-PAH also increasing the number of necrotic cells (Fig. 3b). At 30 μg/ml, all particles increased cell death, with the PAH-coated particles inducing both necrosis and apoptosis, whereas P90 only increased necrosis (Fig. 3a and b). Of note, necrotic and apoptotic cells represented no more than 2% of all counted airway epithelial cells, suggesting that this small amount of damage might not be detectable with common assays.

## Discussion

Our results suggest that the biological effect of PAH-coating is a combination of specific surface area and PAH-specific effects.

In our study, we used four CBNP: P90, P90 coated with BaP (P90-BaP), P90 coated with 9NA (P90-9NA), and, to model nanoparticles that acquire PAH during synthesis, a soot derived from acetylene combustion that contains various PAHs (AS-PAH). This allowed us to examine how PAH coating changes the behavior of the P90 nanoparticle in various test systems. These particles were tested in vitro, ex vivo and in vivo.

### Particle characteristics

Nanoparticle reactivity is determined by the surface reactivity, including the nanoparticle characteristics: size, shape, solubility, surface area, and chemical composition [2, 5, 8, 9, 51, 52]. All particles that we used had similar primary particle size, shape, and hydrodynamic diameters and ζ-potential in medium. An equal solubility was accomplished by adding protein, resulting in negative ζ-potential at physiological pH-value. Thus, any differences observed are attributable to the surface area or surface chemical composition of the CBNP. Coating of P90 with either PAH reduced its surface area from 302 m^2^/g to 91 m^2^/g; since the effects of P90 in biological systems are attributed to its high surface area, this reduction would be expected to reduce the observed biological effects [1, 10].

BaP is considered a tumorigenic reference PAH, with well characterized effects, including induction of ROS formation, IL-8 release and apoptosis [6, 15, 53–57]. It is metabolized by Cyp1A1 and 1B1, and has been shown to upregulate these enzymes both on mRNA and protein levels [23, 58]. Thus, increases in *Cyp1a1* and *1b1* mRNA indicate that this PAH is bioavailable in cells. PAH with 4 and more aromatic rings and specific structural features (Bay- or Fjord-region) are strong Cyp inducers and considered to be tumorigenic [15, 59]. Since 9NA is composed of only three aromatic rings, it is less toxic than BaP and considered as weak Cyp inducer [15, 43] as confirmed by our *Cyp1a1* and *Cyp1b1* mRNA expression results.

Since it is possible that coating of CBNP may result in a different distribution of PAH compared to particles that acquire PAH during synthesis, we also used AS-PAH synthesized from acetylene combustion as a test compound [44, 60]. Since AS-PAH has a similar surface area, shape, size and solubility compared to PAH-coated P90, the different effects of these particles observed in the biological systems are likely the result of its different PAH composition [61, 62]. The AS-PAH that we synthesized contained a mixture of at least 17 PAH with 2–7 aromatic rings that could be removed from the particle with organic solvents, indicating that these PAH are on the surface of the particle. Several of the identified PAH are predicted to be metabolized by Cyp1A1 and 1B1 and induce their mRNA. Thus, by determining the mRNA expression of both enzymes allowed us to determine if the PAHs from AS-PAH are bioavailable. The presence of PAHs with 5 or more aromatic rings predict that, compared to BaP, the PAH mixture of AS-PAH would be more toxic [15, 63]. For example, compared with BaP the 5-ring component cyclopenta[cd]pyrene induced the same mutation mechanism [64], a stronger lung tumorigenic response in A/J mice [65] and was 6.9 ± 4.2-fold more mutagenic in vitro [43]. In addition, PAH-mixtures are often more toxic than would be expected by simple addition of the individual PAH toxicities [65, 66]. Thus, when not bound to CBNP the PAH mixture from AS-PAH is likely to be more toxic compared to BaP. Since the relative in vivo effects of PAHs depend on their bioavailability, their clinical impact cannot be fully predicted without appropriately designed and conducted studies, such as those we report here.

When predicting the behavior of PAH-coated versus uncoated nanoparticles in biological systems, three different scenarios are possible. First, the reduction of the surface area could reduce the biological effects of CBNP, and differences in the PAHs would therefore not have an impact. Second, the PAH content could increase the biological effects of CBNP due to the toxicity of PAH – and so irrespective of the particle’s surface area. A third possibility is that toxicity could be decreased in some cell types due to the reduced surface area, but increased in other cell types due to PAH toxicity.

### Biological effects of uncoated and PAH-coated CBNP

Supporting the hypothesis that a reduced surface area reduces the biological effects of CBNP [1, 10, 67], the coating of P90 with BaP prevented the P90-induced increase of the neutrophil attracting cytokine *IL8* mRNA in A549 cells. As the high surface area of P90 is strongly associated with induction of oxidative stress, the reduced biological effect can be explained by the reduced ROS release that we observed with P90-BaP and P90-9NA in A549 cells compared to uncoated P90. This reduced biological effect is also supported by our observation that P90, and not P90-BaP, led to a transient increase in granulocyte influx in the in vivo experiment. This transient increase in granulocytes without changes of total cell number has previously been observed [68, 69]. The mechanism for the reduced number of macrophages is not known and would be an interesting area for future research. However, these results clearly demonstrate that surface area can be an important factor of CBNP effects in biological systems.

However, other results support the theory that PAH can increase the effects of CBNP [29]. Firstly, we found that P90-BaP, P90-9NA and AS-PAH induced apoptosis in tracheal epithelial cells ex vivo, whereas P90 did not. This is consistent with previous reports describing apoptosis induction by PAH [54, 56, 57, 70]. Secondly, PAH-coated P90 and AS-PAH strongly induced *Cyp* mRNA expression. *Cyp1a1* and *Cyp1b1* mRNA are known to be induced by PAH in a subgroup of airway epithelial cells [71], and so their induction in both the trachea and intrapulmonary airways demonstrate that the PAH are biologically active. Interestingly, the assumed differences between P90-9NA, P90-BaP and AS-PAH are also reflected in the level of induction of *Cyp1a1* and *Cyp1b1* mRNA, in that P90-9NA resulted in the least induction, followed by P90-BaP, with the strongest induction by AS-PAH. Finally, AS-PAH was the only CBNP to reduce TEER in vitro, to increase BAL granulocyte content 14 days after the last application, and induce both histological changes in vivo and inflammatory mediator mRNA expression (*KC* and *IL-6*) ex vivo. Furthermore, AS-PAH had the highest apoptosis rate in tracheal epithelium ex vivo.

Our data therefore suggest that the overall biological impact of a PAH depends on the relative contribution of surface area reduction and PAH-induced toxicity, that is, in turn, influenced by the biological system. This is exemplified by cilia-driven particle transport speed, which is influenced by ciliary beat frequency, mucus release and epithelial cell death.

In the ex vivo trachea, P90 was the only particle to form aggregates. These aggregates bound to ciliated cells and increased ciliary beat frequency. Furthermore, microscopically visible nanoparticle agglomerates have been shown to increase ciliary beat frequency in the murine trachea [72]. A similar effect has been observed in the bovine trachea where polystyrene particles >200 nm in diameter that attach to cilia increase ciliary beat frequency due to mechanical stimulation of axonema possibly involving second messengers such as cAMP or cGMP [73], both of which are known to increase ciliary beat frequency [74]. In addition, mechanical stimulation of cilia triggers Ca^2+^ influx, with a consequent increase in ciliary beat frequency [75]. Coating of P90 with PAHs prevented aggregate formation and consequently neither P90-BaP nor P90-9NA impact ciliary beat frequency. Thus, this shows that PAH coating of P90 reduced its effects on a biological system by changing its surface properties and not by a specific PAH toxicity. However, we detected an increase in epithelial cell apoptosis after application of P90-BaP and P90-9NA compared to P90. This increase in apoptosis together with the induction of Cyp enzymes can directly be ascribed to PAH toxicity [70].

AS-PAH increased ciliary beat frequency without forming microscopically visible agglomerates. Interestingly, fluoranthene, a PAH that we detected on the surface of AS-PAH, has previously been shown to induce a persistent increase in Ca^2+^ in Calu-3 cells, whereas BaP and anthracene did not [76]. Since an increase in intracellular Ca^2+^ is known to increase ciliary beat frequency [74], it is possible that fluoranthene is directly responsible for the increased ciliary beat frequency. Despite increasing ciliary beat frequency there was a marked reduction in particle transport speed, possibly due to increased apoptosis and mucus release.

Previous research has shown that despite being bound to the surface of CBNP, PAHs can be bioavailable [77]. Our data confirm that PAH bound to CBNP exerts biological effects in epithelial cells, as demonstrated by *Cyp1a1* mRNA induction. *Cyp1a1* mRNA expression is a very sensitive parameter for detecting biologically active PAHs [78]. The induction of *Cyp1a1* mRNA is triggered by binding of PAH to the aryl hydrocarbon receptor (AhR) that then translocates into the cell nucleus, and interacts with AhR nuclear translocator [79]. This complex then induces *Cyp1a1* mRNA expression by binding to xenobiotic response elements [79]. In low concentrations, AhR activation suppresses the activity of the transcription factor p65 [80], which is known to induce the mRNA expression of *IL-6* and *IL-8* [81, 82]. We also observed a small but significant decrease in *IL-6* mRNA expression in the epithelium of explanted tracheae, possibly due to AhR activation. At higher concentrations, PAHs can lead to *IL-6* and *IL-8* mRNA expression [80] – as we observed with AS-PAH. In general, AS-PAH had more effects than the PAH-coated CBNP, probably due to the composition of PAH present on the particle’s surface. As discussed above, the PAH fluoranthene induces prolonged increase in intracellular Ca^2+^ in Calu-3, whereas BaP and anthracene do not [76]. Yamaguchi et al. also observed an increase of intracellular Ca^2+^ that was independent from AhR [83], indicating that PAH have additional activities apart from the classical AhR pathway. Identification of the specific PAH and/or PAH mixture that is responsible for the increased effects of AS-PAH would require additional research, although fluoranthene and compounds such as cyclopenta[cd]pyrene are possible candidates.

We have shown that AS-PAH exerts more toxic effects than P90 despite its reduced surface area. We attribute this to the composition of the surface PAHs. The relative toxicity of P90-9NA and P90-BaP, and the resultant biological effect, depended on the test system used.

### Choice of test systems

In general, cell lines can give valuable information if the test substance is interfering with basal cell functions present in all cells. However, if cell lines lack specific pathways that are important for toxic effects, their value for predicting in vivo toxicity is limited [84].

The prediction of an in vivo effect based on results obtained in a single cell line is made more challenging, since both changes in surface area and direct PAH toxicity impact the overall effect of particles. This may explain why we had different results in terms of *IL-8* mRNA production in different cell lines. The reason for these differences was most likely that some cell lines were more sensitive to the toxic effects of the PAHs, whereas others were more sensitive to changes in surface area. An in vivo experiment in which all target cell types are present is therefore in principle better suited to detect effects [84]. However, in a single in vivo experiment, only a limited number of parameters can be determined. The readouts predetermined in the OECD experiment focus on inflammation and cell death. They do not address effects on mucociliary clearance. It should be noted, however that sustained impairment of mucociliary clearance can have substantial health effects [85–88] that are missed in animal models by focusing solely on inflammation and cell death. In principle, in vivo experiments can be designed to also include these parameters. However, this would be time consuming and cost intensive. The strength of short-term organ culture models is that they can be adapted to screen for specific organ functions that are not routinely examined in standardized in vivo experiments. This was demonstrated by using our ex vivo trachea model that allowed examining the complex interplay between mucus production, ciliary beating and cell death [72, 89].

## Conclusions

Using a range of different test systems our results demonstrate that the biological effect of CBNP is determined by a combination of specific surface area and the composition of the surface-bound PAH, and varies in different target cells. Specifically, AS-PAH exerted more toxic effects than P90 despite its reduced surface area. We attribute this to the composition of the surface PAHs. However, the relative toxicity of P90-9NA and P90-BaP depended on the test system used.

## Additional files

Additional file 1: Electron microscopic images of P90 and AS-PAH. (PDF 428 kb)

Additional file 2: AS-PAH exhibited a less PAH content and a PAH mix compared to the coated P90 nanoparticles. (PDF 308 kb)

Additional file 3: Primer pairs and probes used by quantitative real time RT-PCR. (PDF 75 kb)

Additional file 4: Staining of necrotic and apoptotic cells in the epithelial layer. (PDF 295 kb)

Additional file 5: Supplemental material and methods. (DOCX 31 kb)

Additional file 6: CBNP did not affect the viability of epithelial cells, but P90-BaP and AS-PAH induced cytokine release in vitro. (PDF 91 kb)

Additional file 7: CBNP aerosol concentration and results of Marple impactor measurements used by nose-only inhalation experiments. (PDF 56 kb)

Additional file 8: P90-BaP and AS-PAH increased the lung wet weights after nose-only inhalation. (PDF 67 kb)

Additional file 9: CBNP did not increase enzyme nor total protein levels in BAL and did not induce oxidative DNA-damage in BAL cells after nose-only inhalation. (PDF 80 kb)

Additional file 10: Only AS-PAH induced exposure-related histological alteration in lung tissue after nose-only inhalation. (PDF 72 kb)

Additional file 11: CBNP did not induce oxidative stress or a pro-inflammatory response in intrapulmonary airways. (PDF 106 kb)

Additional file 12: P90 agglomerates increased ciliary beat frequency and released mucus impaired particle transport speed. (PDF 573 kb)

Additional file 15: P90 did not cause agglomerates at 1 μg/ml, but released mucus impaired particle transport. (PDF 1144 kb)

Additional file 16: P90-BaP induced cell death. (PDF 707 kb)

Additional file 17: P90-9NA induced cell death at the higher concentration. (PDF 641 kb)

Additional file 18: P90 induced mucus release. (PDF 1021 kb)

Additional file 19: AS-PAH induced mucus release and cell death. (PDF 639 kb)

Additional file 20: No CBNP increased ciliary beat frequency at 30 μg/ml. (PDF 5594 kb)
