# Supplementary material for: Biological effects of carbon black nanoparticles are changed by surface coating with polycyclic aromatic hydrocarbons
Source: Part Fibre Toxicol. 2017 Mar 21;14:8. doi: 10.1186/s12989-017-0189-1 (PMC5361723; doi:10.1186/s12989-017-0189-1)
Supplement: Supplementary file 3 — Electron microscopic images of P90 and AS-PAH. (PDF 428 kb) [file 12989_2017_189_MOESM1_ESM.pdf]

Additional file 1

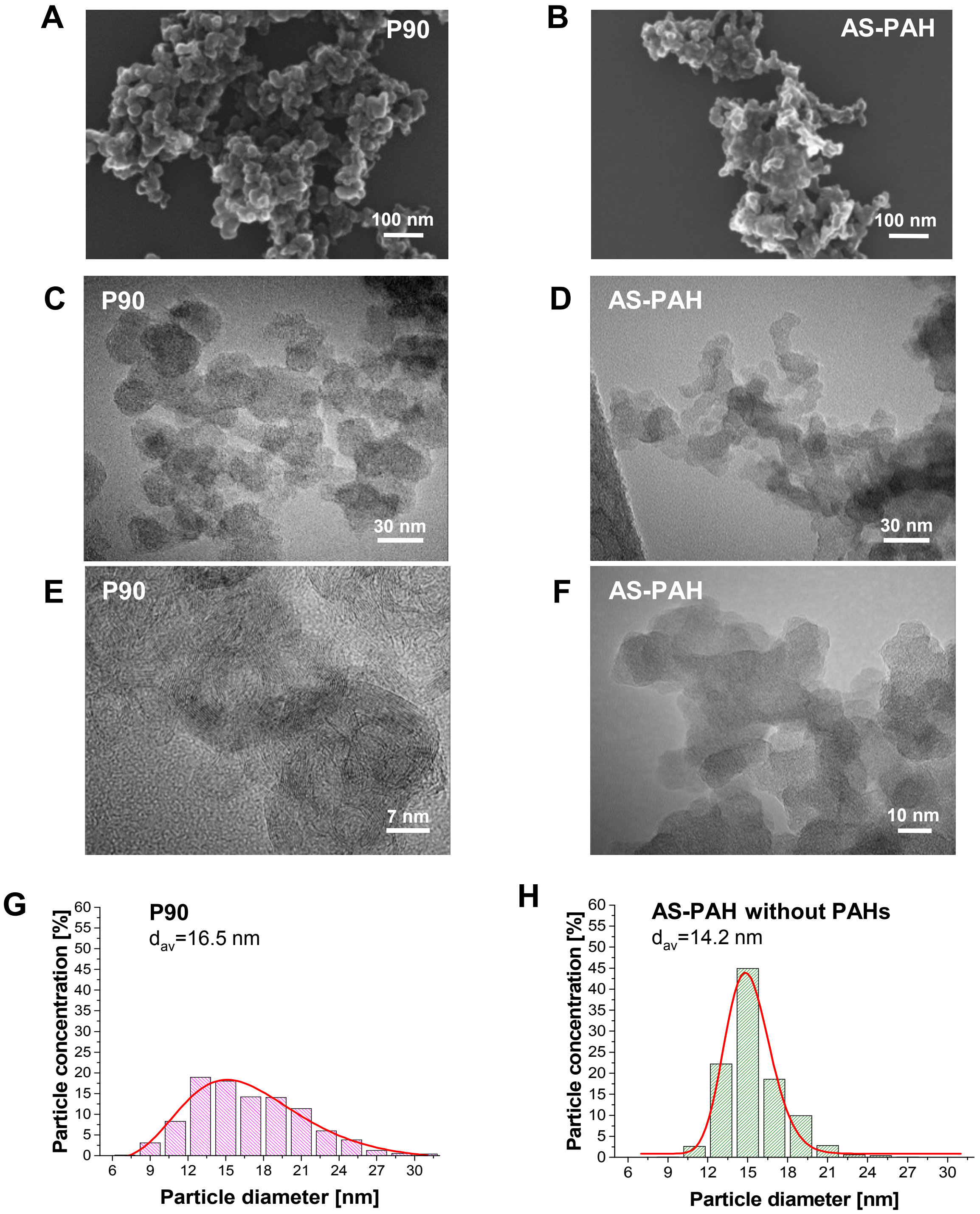

Electron microscopic images of P90 and AS-PAH

A,**B**) show representative scanning electron microscopic images of P90 (**A**) and PAH-depleted AS-PAH (**B**). **C**-**F**) show representative transmission electron microscopic images of P90 (**C**,**E**) and PAH-depleted AS-PAH (**D**,**F**). **G**,**H**) Diagrams display the particle size distributions of P90 (**G**) and AS-PAH without PAH (**H**).
