# Supplementary material for: Biological effects of carbon black nanoparticles are changed by surface coating with polycyclic aromatic hydrocarbons
Source: Part Fibre Toxicol. 2017 Mar 21;14:8. doi: 10.1186/s12989-017-0189-1 (PMC5361723; doi:10.1186/s12989-017-0189-1)
Supplement: Supplementary file 4 — AS-PAH exhibited a less PAH content and a PAH mix compared to the coated P90 nanoparticles. (PDF 308 kb) [file 12989_2017_189_MOESM2_ESM.pdf]

Additional file 2

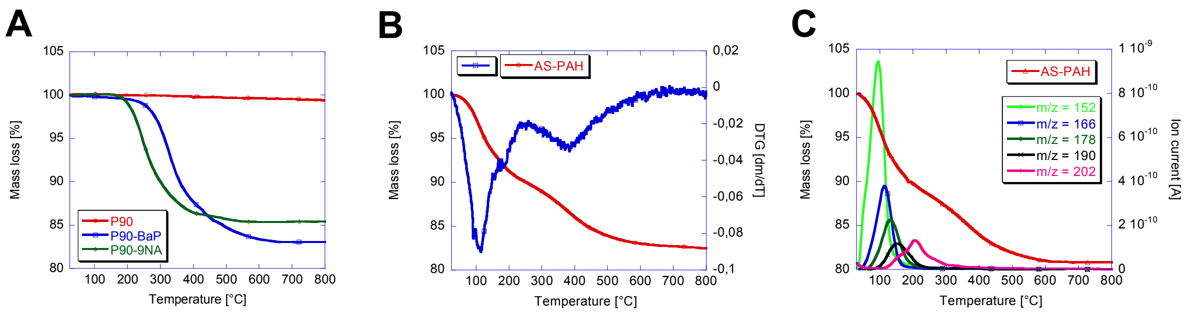

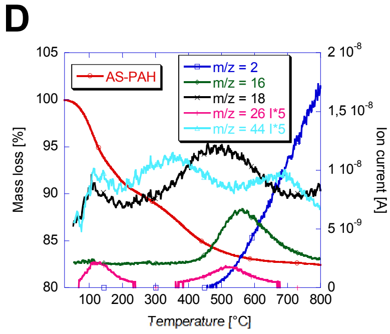

E

m/z	Name	sum formula
152	Biphenylene	$C_{12}H_8$
152	Acenaphthylene	$C_{12}H_8$
166	Fluorene	$C_{13}H_{10}$
178	Phenanthrene	$C_{14}H_{10}$
178	Anthracene	$C_{14}H_{10}$
190	4H-Cyclopenta[def]phenanthrene	$C_{15}H_{10}$
202	Fluoranthene	$C_{16}H_{10}$
202	Pyrene	$C_{16}H_{10}$

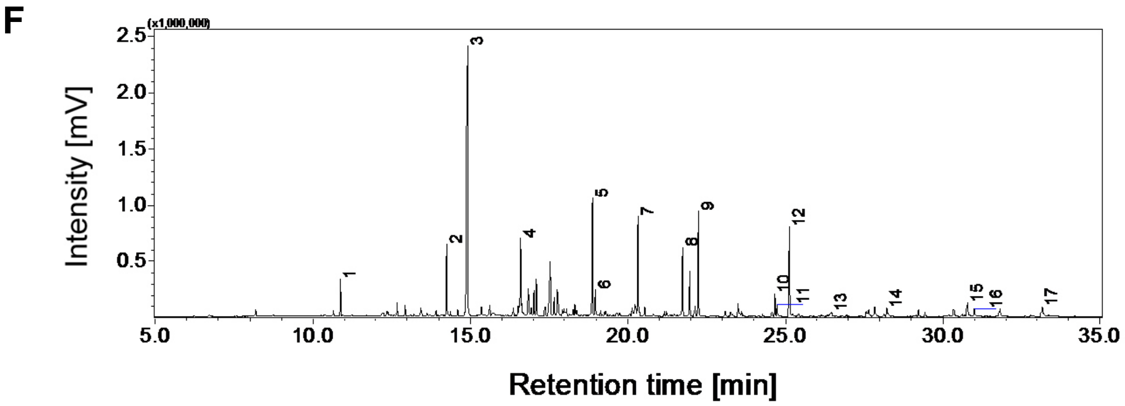

AS-PAH exhibited a less PAH content and a PAH mix compared to the coated P90 nanoparticles.

A) Representative thermograms of P90 and P90 coated with BaP or 9NA. One representative thermogram of AS-PAH is shown as the red line in (**B**). The blue line in **B** represents the derivative thermogravimetric (DTG) results of the delta-mass-to-delta-temperature ratio, illustrating the two-stage thermal desorption. Figures **C** and **D** show thermograms and mass-spectra curves of AS-PAH detected for the range m/z=100–250 (**C**) and m/z=1–100 (**D**). **C**) In the first stage, PAHs were predominantly detected. **D**) The fragments m/z=26 and 44 in the first stage were PAH fragments or acetylene and carbon dioxide. In the second stage, a release of hydrogen (m/z=2), methane (m/z=16), water (m/z=18), acetylene (m/z=26) and carbon dioxide (m/z=44) occurred. Possible PAH candidates are represented in (**E**). Figure **F** shows a representative GC/MS chromatogram of PAHs from an AS-PAH sample. m/z=mass-to-charge ratio
