# Supplementary material for: Biological effects of carbon black nanoparticles are changed by surface coating with polycyclic aromatic hydrocarbons
Source: Part Fibre Toxicol. 2017 Mar 21;14:8. doi: 10.1186/s12989-017-0189-1 (PMC5361723; doi:10.1186/s12989-017-0189-1)
Supplement: Supplementary file 5 — Primer pairs and probes used by quantitative real time RT-PCR. (PDF 75 kb) [file 12989_2017_189_MOESM3_ESM.pdf]

Additional file 3

Primer pairs used by quantitative real time RT-PCR

Target	NM number	Sequence Primer sense (5´-3´)	Sequence Primer antisense (5´-3´)	Used for
IL-8	000584.3	TGCCAAGGAGTGCTAAAG	CTCCACAACCCTCTGCAC	A549 cells, 16HBE14o- cells
IL-8	000584.3	ACCACACTGCGCCAACAC	CTTCTCCACAACCCTCTGCAC	Calu-3 cells
HPRT1	000194.2	GACCAGTCAACAGGGGACAT	CTTGCGACCTTGACCATCTT	A549 cells, 16HBE14o- cells
HPRT1	000194.2	GTCAGGCAGTATAATCCAAAGA	GGGCATATCCTACAACAAACT	Calu-3 cells
Cyp1a1	009992.4	TTCCTGTCCTCCGTTACCTG	CCTGTCCTGACAATGCTCAA	Mouse airways
Cyp1b1	009994.1	TTACGGACATCTTCGGAGCC	AACCTGGTCCAACTCAGCC	Mouse airways
Gpx3	008161.3	CAGGCGAGAACTCGGAGATA	AGCGGATGTCATGGATCTTC	Mouse airways
Gr	010344.4	TCGGAATTCATGCACGATCAC	TGTTCAGGCGGCTCACATAG	Mouse airways
IL-6	031168.1	CTCCCAACAGACCTGTCTATAC	GTGCATCATCGTTGTTCATAC	Mouse airways
KC (Cxcl1)	008176.3	CAGACCATGGCTGGGATTC	GAACCAAGGGAGCTTCAG	Mouse airways
Rpl32	172086.2	AAAATTAAGCGAAACTGGCG	ATTGTGGACCAGGAACTTGC	Mouse airways

Probes used by quantitative real time RT-PCR

Target	NM number	Sequence TaqMan FAM-TAMRA (5´-3´)
Cyp1a1	009992.4	CTTCCCTGGATGCCTTCAAGGACTTGAATG
Cyp1b1	009994.1	CCTTTCCACCGCGCTGCTGTGGCTG
Gpx3	008161.3	CAAGTATGTTCGACCAGGTGGGGGCTTTG
Gr	010344.4	GGCTTTCAAAGCTGTGAGGGTAAATTCAGTTGG
IL-6	031168.1	TGCCATTGCACAACTCTTTTCTCATTTCCACG
KC (Cxcl1)	008176.3	CCTCGCGACCATTCTTGAGTGTGGCTATGAC
Rpl32	172086.2	CATTGACAACAGGGTGCGGAGAAGGTTC
