# Supplementary material for: Biological effects of carbon black nanoparticles are changed by surface coating with polycyclic aromatic hydrocarbons
Source: Part Fibre Toxicol. 2017 Mar 21;14:8. doi: 10.1186/s12989-017-0189-1 (PMC5361723; doi:10.1186/s12989-017-0189-1)
Supplement: Supplementary file 6 — Staining of necrotic and apoptotic cells in the epithelial layer. (PDF 295 kb) [file 12989_2017_189_MOESM4_ESM.pdf]

Additional file 4

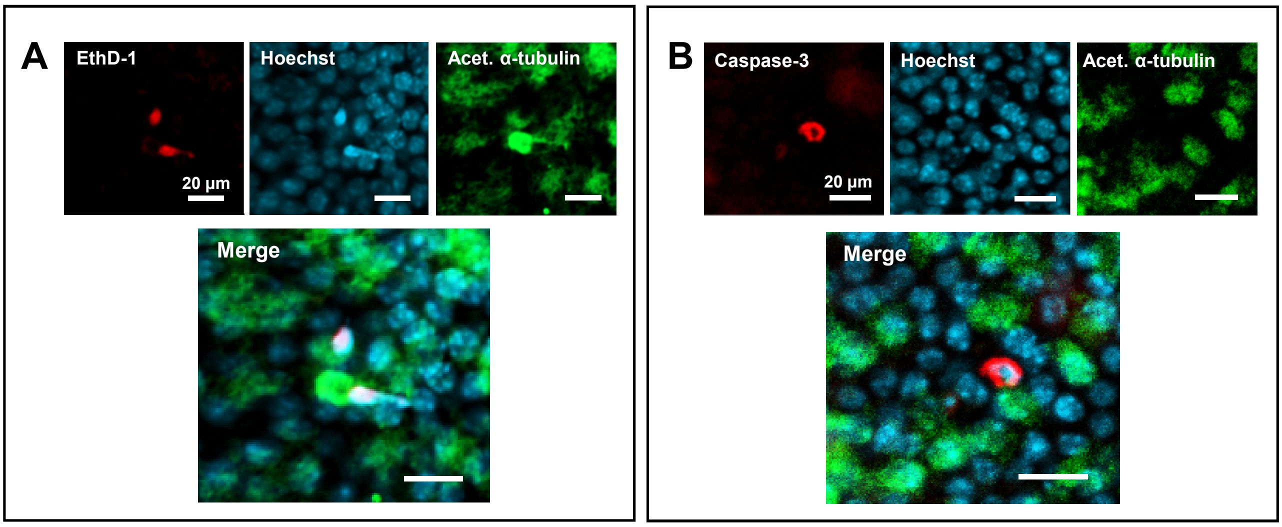

Staining of necrotic and apoptotic cells in the epithelial layer

Images show necrotic cells stained with ethidium homodimer-1 (EthD1, red, **A**) and a apoptotic cell stained with anti-cleaved caspase-3 antibody (red, **B**). The cell nuclei were stained with Hoechst dye (blue) and ciliated cells were stained with a anti-acetylated α-tubulin antibody (green).
