# Supplementary material for: Biological effects of carbon black nanoparticles are changed by surface coating with polycyclic aromatic hydrocarbons
Source: Part Fibre Toxicol. 2017 Mar 21;14:8. doi: 10.1186/s12989-017-0189-1 (PMC5361723; doi:10.1186/s12989-017-0189-1)
Supplement: Supplementary file 7 — Supplemental material and methods. (DOCX 31 kb) [file 12989_2017_189_MOESM5_ESM.docx]

**Additional file: Material and Methods**

### **Endotoxin tests**

CBNP suspensions were tested for endotoxins using the CROMO-LAL assay (Associates of Cape Cod Inc., East Falmouth, MA, USA) according to the manufacturer´s protocol. The limit of detection was 0.005 EU/ml comparable to 0.5 pg/ml endotoxin of E. coli Type 055:B5. The suspensions were only used for experiments if no endotoxins were detected.

***In vitro* experiments**

**Cytokine release**

Cytokine levels were measured in supernatants of Calu-3 cells used form RNA isolation and following quantitative RT-PCR. CXCL8 (IL-8) and IL-6 levels were assessed with commercial ELISA kit systems (both Thermo Fisher Scientific Inc., Waltham, USA) according to manufacturer's instructions.

**WST-8 cell viability assay**

A549 cells, Calu-3 cells and 16HBE14o- cells were seeded in 48 well plates (Costar, Corning, NY, USA) at 2.4×10^4^, at 1.3×10^5^ cells and 4×10^4^ cells, respectively. After reaching a confluency of ≥90%, the cells were exposed to 10 µg/ml and 50 µg/ml CBNP for 24 hours at 37°C and 5% CO_2_. After 24 hours, the suspensions were removed from the cells and 250 µl fresh, pre-warmed medium and 25 µl WST-8 (2-(2-methoxy-4-nitrophenyl)-3-(4-nitrophenyl)-5-(2,4-disulfophenyl)-2H-tetrazolium, monosodium salt; CCK-8 Solution, Sigma Aldrich, Schnelldorf, Deutschland) were added into each well and incubated for 2 hours at 37°C and 5% CO_2_. WST-8 produces a water-soluble formazan dye, whose amount produced is directly proportional to the number of living cells. The supernatants were removed from the cells and measured at 450 nm (ref. 650 nm) in an Epoch microplate spectrophotometer (BioTek Instruments GmbH, Friedrichshall, Germany).

**Nose-only inhalation study in rats**

**CBNP aerosol generation and exposure of rats**

The particulate CBNP aerosols were generated by dispersing the dry CBNP powder. Dispersion was achieved by a feeding system and a high-pressure, high-velocity pressurized air dispersion nozzle developed by Fraunhofer ITEM [1].

The aerosol was generated by a high-pressure pneumatic disperser, which was fed with P90, P90-BaP and AS-PAH under computerized control, i.e., with a feedback loop to the actual aerosol concentrations measured by an aerosol photometer. The photometer gives a scattering light signal, which is proportional to the particle concentration, if the particle size distribution is constant. The ratio between photometer signal and actual concentration was determined throughout the study by comparing to gravimetric concentrations. The aerosol was given to the rats by a flow-past nose-only inhalation exposure system. The mass median aerodynamic diameter (MMAD) was determined twice by a cascade impactor (Marple impactor). MMADs were measured once per exposure group before the exposure period and once throughout the exposure period. The critical value for respirability of an aerosol in rats is 3 µm. Filter samples of the aerosols were taken at least twice per week to control the aerosol concentrations and to calibrate the aerosol photometers. For exposure, the rats were restrained in acrylic tubes with a flexible stopper.

**Endpoints**

*Bronchoalveolar lavage and determination of biochemical indicators*

After centrifugation of the lavage fluid, biochemical indicators relevant for diagnosis of lung damage were determined in the supernatant (i.e. lactic dehydrogenase [LDH], β-glucuronidase, total protein) by using specific assays.

*Oxidative Comet-Assay in BAL cells*

DNA-strand breaks and oxidative DNA damage (8-OH-dG) were analyzed in BAL cells of 5 animals per dose group on day 1 (24 hours after the last exposure) and day 14 post exposure using human 8-hydroxyguanine DNA-glycosylase 1 (hOGG1)-modified alkaline comet assay [2], based on the alkaline version of the comet assay [3, 4]. KBrO_3_-positive controls were used to assess both activity of the used hOGG1 enzyme batch and appropriate performance of cell isolation, slide preparation, electrophoresis, and staining. BAL cells were derived from the same animals as used for determination of BAL parameters.

Two aliquots of each BAL cell suspension (a minimum of 20,000 cells in a volume of 1 ml) were centrifuged, resuspended in pre-heated 0.75% low melting agarose, applied to agarose pre-coated slides, and lysed overnight at 4°C to liberate the DNA. One of the two slides per animal was subsequently incubated for 10 min with 0.16 U/ml of hOGG1 to detect oxidative DNA-damage. The other slides received enzyme buffer only. DNA-unwinding and electrophoresis was done on ice in 4°C cold electrophoresis buffer (300 mM NaOH, 1 mM EDTA, pH >13). DNA was stained with ethidium bromide and analyzed using the Comet Assay III Software (Perceptive Instruments Ltd., Bury St. Edmunds, UK). As the main endpoint, the tail intensity (TI) of at least 100 nuclei per animal and slide treatment (i.e., with or without hOGG1 incubation) was determined (meaning in total at least 500 nuclei per group and slide treatment). TI is a direct measure (total fraction of DNA in the tail and thus damaged DNA) that can be standardized among various studies, and bears linear relationship to break frequency, so enables discrimination of DNA damage over the widest possible range of (0-100% DNA in tail). An increase in TI on the hOGG1-treated slides, as compared to the slides treated with enzyme buffer only, is indicative for the occurrence of the oxidative base lesion 8-OHdG.

For estimation of the viability and integrity of the BAL cell suspensions on an animal basis and to avoid artefacts due to cytotoxicity of the different CBNPs and KBrO_3_ reference, so called “hedgehogs” (comets with small or non-existing heads and large diffuse tails) were excluded from main analysis, but percent “hedgehogs” were analyzed separately as a parameter for cytotoxicity.

Besides the hOGG1-modified comet assay, BAL parameters had to be determined from the same BAL cell preparations. Since this meant that a reduced cell number was available for both of these assays, we generated only the two slides per animal needed for a single evaluation of DNA-damage in the hOGG1-modified comet assay. No replicate slides could be used, as originally recommended by [5]. To compensate for the number of analyzed nuclei, 100 nuclei per slide were finally analyzed instead of 50 nuclei per replicate slide to at least follow the recommendation of 100 nuclei per animal [5].

#### *Histopathology*

On days 1 and 14 post treatment, histopathology of the lungs, including bronchi and lung-associated lymph nodes (LALN), was conducted on 5 animals per group. Lungs were inflated with 10% neutral-buffered formalin, fixed by immersion, and embedded in paraffin, sectioned and stained with hematoxylin and eosin (H&E). CBNP-induced histological changes were semi-quantitatively scored, using a grading system that considered the severity, number and size of histopathological lesions. The severity of each lesion was graded on a scale of very slight to very severe, together with the approximate fraction of the organ/tissue or organ structure involved, from very slight (or minimal), when 1-5 % was involved, through to very severe (or massive), with 75-100 % involvement.

**Statistics**

For evaluation of the hOGG1-modified comet assay the Student’s t-test for unpaired values, two-sided, was used for comparison of the different treatment groups with the clean air controls. Student’s t-test for paired values, two-sided, was used for comparing the slides with hOGG1-treatment with the slides, which received enzyme buffer only, to estimate occurrence of the oxidative base-modification 8-OHdG.

The statistical evaluation of the histopathological findings was done with the two-tailed Fisher test by the PROVANTIS software system (Insteam group, Staffordshire, UK).

**Experiments with explanted mouse intrapulmonary airways**

**Particle suspension in cell culture medium**

CBNPs were suspended in water (100 µg/ml) as described above, and then mixed 1:1 with 2x F-12 Medium with 0.2 mM L-Cysteine, 10 mM HEPES and 14 mM NaHCO_3_ (Nutrient mix; Life Technologies GmbH, Darmstadt, Germany) for exposure of intrapulmonary airways.

For experiments, nanoparticles were diluted further to final concentrations of 10 µg/ml in F12 medium with 2.5 mg/ml insulin, 12.5 mg/ml EGF, 50 µg/ml bovine hypothalamus extract, 1 mM hydrocortisone, 2.5 mg/ml transferrin, 1% Pen/Strep, 50 mg/ml gentamicin, 2 mM retinol for incubation of intrapulmonary airways.

**Animals**

We used 8 to 12 weeks old female Balb/c mice (Charles River Laboratories, Sulzfeld, Germany). Mice were euthanized by cervical dislocation under anesthesia after intraperitoneal injection of 300 µl 6% ketamine (CP-Pharma Handelsgesellschaft mbH, Burgdorf, Germany), 0.22% xylazine (Rompun^®^; Bayer HealthCare LLC, Animal Health, Leverkusen, Germany) in PBS for preparation of intrapulmonary airways. The *ex vivo* studies on intrapulmonary bronchioles were approved by the Ministerium für Landwirtschaft, Umwelt und ländliche Räume des Landes Schleswig-Holstein.

**Preparation and culture conditions of intrapulmonary airways**

Distal and proximal intrapulmonary airways were dissected from the left lobe of explanted, agarose-filled lungs (method published in [6]) and transferred to transwells of Corning Costar cell culture plates (Corning Incorporated, NY, USA) with 0.4 µm PET Milipore cell culture inserts (Merck Milipore, MA, USA). Bronchioles were incubated in transwells, one proximal airway per insert or four distal airways per insert, with a total volume of 1.5 ml supplemented F12-medium with or without CBNP suspension (1ml per well and 500 µl per insert) at 37°C with 5% CO_2_ for 24 hours.

**Qualitative RT-PCR**

After incubation with CBNP medium suspension or medium alone, the proximal and distal airways were transferred in the lysis buffer of RNeasy^®^Mirco Kit (Qiagen GmbH, Hilden, Germany) and homogenized. RNA isolation and DNA digestion were performed according to the manufacturer’s protocols. The isolated mRNA was transcribed to cDNA using Maxima First Strand cDNA synthesis kit (Fisher Scientific-Germany, Schwerte, Germany).

Real time RT-PCR was performed with Roche Light Cycler 480 Sybr green I Master (Roche Diagnostics Deutschland GmbH, Mannheim, Germany) according to the manufacturer´s protocol. The following PCR program was used: one cycle at 95°C for 10 min followed by 45 cycles touch-down 63°C to 58°C for 10 s with 0.5°C/s and 5 s (*RPL-32*, *KC*) or 8 s (*Cyp1a1*, *IL-6*) at 72°C. Afterwards a melting curve was generated. Changes in the transcript levels were determined by calculating the n-fold difference in cycle threshold to the respective negative control by normalization. The primers used for quantitative real time RT-PCR are represented in Additional file 4. All primers span an exon-exon region on specific mRNA.

**Statistical analysis**

Data are presented as mean ± SEM. All ex vivo experiments were carried out at least three times. The data sets were analyzed using Mann-Whitney U test in GraphPad Prism 5 (GraphPad Software, Inc., La Jolla, CA, USA). P values < 0.05 were considered as statistically significant

**Experiments with explanted mouse trachea**

**Macroscopic analysis**

After incubation, trachea pieces were analyzed using a stereomicroscope (WILD, Heerbrugg, Switzerland) equipped with a digital camera (Casio Exilim EX-FC100; Casio Computer Co., LTD, Tokyo, Japan).

**Scanning electron microscopy**

The trachea samples were fixed in Monti-Graziadei solution (pH 3.5) [7] washed with 0.1 M sodium cacodylate buffer (pH 7.4) and dehydrated in acetone. After critical point drying the samples were sputtered with platinum (Polaron SEM coating system, Polaron Instruments, Lewes, England). The surface of the tracheal epithelium was investigated using a scanning electron microscope (SEM 505, Philips/FEI, Eindhoven, Netherlands).

1. Koch W: Application of aerosols. In Pulmonary Research. Edited by Uhlig S, Taylor AE. Basel-Boston-Berlin: Birkhäuser; 1998

2. Smith CC, O'Donovan MR, Martin EA. hOGG1 recognizes oxidative damage using the comet assay with greater specificity than FPG or ENDOIII. Mutagenesis. 2006;21**:**185-90.

3. Singh NP, Mccoy MT, Tice RR, Schneider EL. A Simple Technique for Quantitation of Low-Levels of DNA Damage in Individual Cells. Exp Cell Res. 1988;175**:**184-91.

4. Tice RR, Agurell E, Anderson D, Burlinson B, Hartmann A, Kobayashi H, et al. Single cell gel/comet assay: guidelines for in vitro and in vivo genetic toxicology testing. Environ Mol Mutagen. 2000;35**:**206-21.

5. Hartmann A, Agurell E, Beevers C, Brendler-Schwaab S, Burlinson B, Clay P, et al. Recommendations for conducting the in vivo alkaline Comet assay. 4th International Comet Assay Workshop. Mutagenesis. 2003;18**:**45-51.

6. Van Winkle LS, Isaac JM, Plopper CG. Repair of naphthalene-injured microdissected airways in vitro. Am J Respir Cell Mol Biol. 1996;15**:**1-8.

7. Monti-Graziadei GA, Graziadei PP. Neurogenesis and neuron regeneration in the olfactory system of mammals. II. Degeneration and reconstitution of the olfactory sensory neurons after axotomy. J Neurocytol. 1979;8**:**197-213.
