# Supplementary material for: Biological effects of carbon black nanoparticles are changed by surface coating with polycyclic aromatic hydrocarbons
Source: Part Fibre Toxicol. 2017 Mar 21;14:8. doi: 10.1186/s12989-017-0189-1 (PMC5361723; doi:10.1186/s12989-017-0189-1)
Supplement: Supplementary file 8 — CBNP did not affect the viability of epithelial cells, but P90-BaP and AS-PAH induced cytokine release in vitro. (PDF 91 kb) [file 12989_2017_189_MOESM6_ESM.pdf]

Additional file 6

A

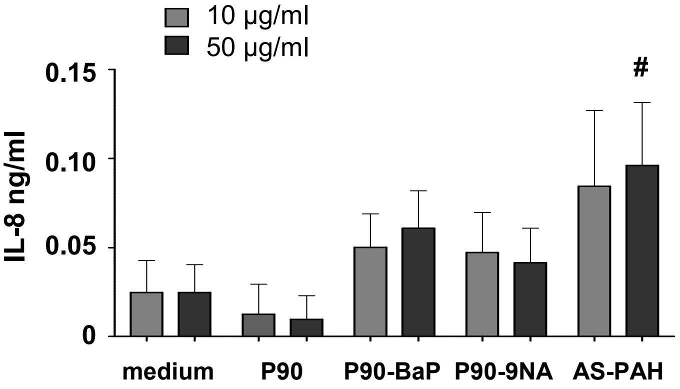

B

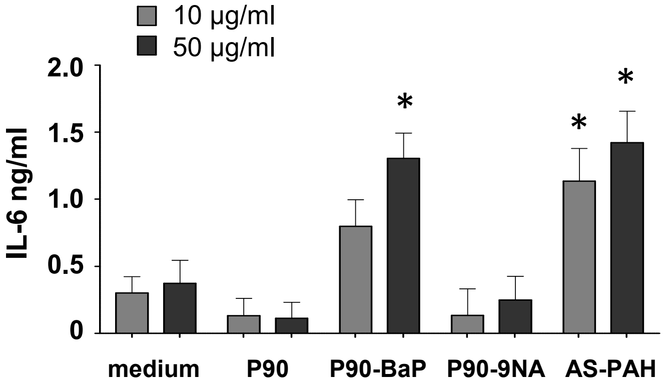

C

	A549		16HBE14o-		Calu-3	
	**10 µg/ml**	**50 µg/ml**	**10 µg/ml**	**50 µg/ml**	**10 µg/ml**	**50 µg/ml**
**P90**	95 ± 5	87 ± 8	97 ± 4	91 ± 7	99 ± 11	90 ± 10
**P90-BaP**	92 ± 2	82 ± 12	85 ± 4	82 ± 6	86 ± 5	82 ± 3
**P90-9NA**	90 ± 4	73 ± 8	80 ± 1	74 ± 3	94 ± 6	82 ± 10
**AS-PAH**	99 ± 8	85 ± 20	95 ± 10	87 ± 6	97 ± 5	92 ± 9

CBNP did not affect the viability of epithelial cells, but P90-BaP and AS-PAH induced cytokine release *in vitro*.

A, **B**) Results of IL-8 (**A**) and IL-6 (**B**) release from Calu-3 cells after 24 hours exposure to 10 µg/ml and 50 µg/m CBNP. Data are mean ± SEM. n=5, except P90-9NA n=3; $^{\#}p<0.05$ CBNP compared to 50 µg/ml P90 and $^{*}p<0.05$ CBNP compared to medium controls analyzed by Mann Whitney U test, respectively.

C) The table shows the results of epithelial cell viability determined by WST-8 assay after 24 hours CBNP-exposure compared to medium control. The data are presented in %. Data are mean ± SEM. n=5
