# Supplementary material for: Biological effects of carbon black nanoparticles are changed by surface coating with polycyclic aromatic hydrocarbons
Source: Part Fibre Toxicol. 2017 Mar 21;14:8. doi: 10.1186/s12989-017-0189-1 (PMC5361723; doi:10.1186/s12989-017-0189-1)
Supplement: Supplementary file 9 — CBNP aerosol concentration and results of Marple impactor measurements used by nose-only inhalation experiments. (PDF 56 kb) [file 12989_2017_189_MOESM7_ESM.pdf]

Additional file 7

A

	P90 (mg/m³)		P90-BaP (mg/m³)		AS-PAH (mg/m³)	
	grav.	phot.	grav.	phot.	grav.	phot.
Mean	5.32	5.31	5.90	5.91	6.06	6.09
SD	0.88	0.88	0.56	0.54	1.41	1.35

B

	P90		P90-BaP		AS-PAH	
	MMAD (µm)	GSD (-)	MMAD (µm)	GSD (-)	MMAD (µm)	GSD (-)
Before exposure	0.63	3.46	2.48	2.83	2.19	2.72
During exposure	0.81	2.81	2.41	3.28	2.87	2.13

CBNP aerosol concentration and results of Marple impactor measurements used by nose-only inhalation experiments

Aerosol concentration measured gravimetrically by filter samples (grav.) and calculated from the photometer signal by using the calibration factor (phot.) are shown in Table **A**. Table **B** shows mass median aerodynamic diameters (MMAD) with geometric standard deviation (GSD).
