# Supplementary material for: Biological effects of carbon black nanoparticles are changed by surface coating with polycyclic aromatic hydrocarbons
Source: Part Fibre Toxicol. 2017 Mar 21;14:8. doi: 10.1186/s12989-017-0189-1 (PMC5361723; doi:10.1186/s12989-017-0189-1)
Supplement: Supplementary file 10 — P90-BaP and AS-PAH increased the lung wet weights after nose-only inhalation. (PDF 67 kb) [file 12989_2017_189_MOESM8_ESM.pdf]

## Additional file 8

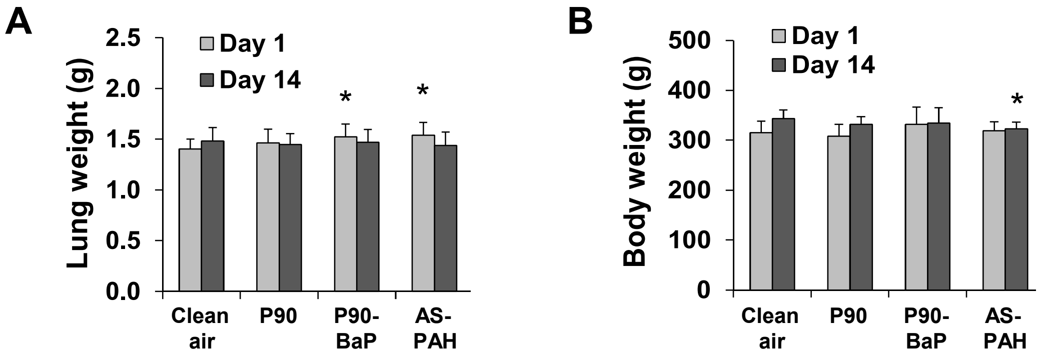


**P90-BaP and AS-PAH increased the lung wet weights after nose-only inhalation.**

The diagrams show the lung wet weights (**A**) and terminal body weights (**B**) after nose-only inhalation of CBNP. Data are mean ± SD; n=10, *p<0.05 CBNP compared to clean air control, analyzed by analysis of variance and Dunnett's test; D1=day 1 post-exposure, D14=day 14 post-exposure
