# Supplementary material for: Biological effects of carbon black nanoparticles are changed by surface coating with polycyclic aromatic hydrocarbons
Source: Part Fibre Toxicol. 2017 Mar 21;14:8. doi: 10.1186/s12989-017-0189-1 (PMC5361723; doi:10.1186/s12989-017-0189-1)
Supplement: Supplementary file 11 — CBNP did not increase enzyme nor total protein levels in BAL and did not induce oxidative DNA-damage in BAL cells after nose-only inhalation. (PDF 80 kb) [file 12989_2017_189_MOESM9_ESM.pdf]

Additional file 9

A

Group	Lactate dehydrogenase (U/l)		β-glucuronidase (U/l)		Total protein (mg/l)	
	D1	D14	D1	D14	D1	D14
**Clean air**	30.8 ± 10.4	24.8 ± 5.6	0.24 ± 0.05	0.26 ± 0.05	99.6 ± 7.8	97.6 ± 17.3
**P90**	43.0 ± 27.1	29.2 ± 11.0	0.34 ± 0.21	0.28 ± 0.08	128.0 ± 34.6	102.0 ± 12.3
**P90-BaP**	34.0 ± 14.3	23.2 ± 2.6	0.20 ± 0.00	0.26 ± 0.05	99.6 ± 10.4	93.6 ± 14.4
**AS-PAH**	55.0 ± 14.7	29.0 ± 4.1	0.38 ± 0.08	0.32 ± 0.08	139.0 ± 21.7	106.0 ± 11.9

B

Group	Tail intensity (%)		Tail intensity (%)	
	Day 1		Day 14	
	-hOGG1	+hOGG1	-hOGG1	+hOGG1
**Clean air**	0.78 ± 0.18	0.66 ± 0.27	1.07 ± 0.74	0.93 ± 0.08
**P90**	**1.70 ± 0.27***	1.59 ± 0.87	1.57 ± 0.90	1.16 ± 0.67
**P90-BaP**	**1.25 ± 0.36***	1.23 ± 0.28	1.18 ± 0.52	1.35 ± 0.88
**AS-PAH**	**1.60 ± 0.39***	1.38 ± 0.43	1.91 ± 0.67	2.32 ± 1.47
**$KBrO_3$ i.p.**	1.97 ± 1.17[a]	4.78 ± 0.28[a]	0.49 ± 0.19	2.82 ± 2.00[#]

CBNP did not increase enzyme nor total protein levels in BAL and did not induce oxidative DNA-damage in BAL cells after nose-only inhalation.

A) The table shows the enzyme and total protein level determined in the BAL after the exposure time on day 1 and day 14 recovery period. Data are mean ± SD; n=5, *p<0.05 CBNP compared to clean air control; D1=day 1 post-exposure, D14=day 14 post-exposure

Results of Comet assay are shown in table (**B**). DNA-strand breaks and oxidative DNA damage (hOGG1) were analyzed in BAL cells on day 1 and 14 of recovery period. Data are mean ± SD; n=5, [a]means of only two animals, because of obvious misinjection, *p<0.05 CBNP compared to clean air control group, [#]p<0.05 data are significantly different from slides without hOGG1 treatment on day 14 post-exposure, i.p.=intraperitoneal exposure to $KBrO_3$.
