# Supplementary material for: Biological effects of carbon black nanoparticles are changed by surface coating with polycyclic aromatic hydrocarbons
Source: Part Fibre Toxicol. 2017 Mar 21;14:8. doi: 10.1186/s12989-017-0189-1 (PMC5361723; doi:10.1186/s12989-017-0189-1)
Supplement: Supplementary file 12 — Only AS-PAH induced exposure-related histological alteration in lung tissue after nose-only inhalation. (PDF 72 kb) [file 12989_2017_189_MOESM10_ESM.pdf]

Additional file 10

	P90		P90-BaP		AS-PAH	
	D1	D14	D1	D14	D1	D14
**Lung**						
Alveolar accumulations of particle-laden macrophages	5	5	5	5	5	5
Interstitial accumulations of particle-laden macrophages	1	0	0	2	4	5
Accumulations of particle-laden macrophages in BALT	4	3	3	3	1	3
Interstitial inflammatory cell infiltration	2	0	3	4	5	5
Interstitial fibrosis	0	0	0	0	2	0
Bronchiolo-alveolar hyperplasia	0	0	0	1	4	3+***2***
**LALN**						
Accumulations of particle-laden macrophages	0	2	0	4	1	5

Only AS-PAH induced exposure-related histological alteration in lung tissue after nose-only inhalation.

The table shows the histopathological findings in lung sections stained with hematoxylin and eosin. The numbers represent the animals with very slight (1-5% involvement) histopathological findings, except the bold italic number which represent the animals with slight findings (6-20% involvement). The clean air control group showed no histopathological changes.

n=5; BALT=bronchus-associated lymphoid tissue; D1=day 1 post-exposure, D14=day 14 post-exposure
