# Supplementary material for: Biological effects of carbon black nanoparticles are changed by surface coating with polycyclic aromatic hydrocarbons
Source: Part Fibre Toxicol. 2017 Mar 21;14:8. doi: 10.1186/s12989-017-0189-1 (PMC5361723; doi:10.1186/s12989-017-0189-1)
Supplement: Supplementary file 13 — CBNP did not induce oxidative stress or a pro-inflammatory response in intrapulmonary airways. (PDF 106 kb) [file 12989_2017_189_MOESM11_ESM.pdf]

Additional file 11

	P90		P90-BaP		P90-9NA		AS-PAH	
	proximal	distal	proximal	distal	proximal	distal	proximal	distal
Gpx3	2.3 ± 0.6	0.9 ± 0.3	3.8 ± 2.5	0.9 ± 0.3	0.7 ± 0.2	1.2 ± 0.6	2.9 ± 0.7	2.6 ± 1.7
Gr	1.2 ± 0.4	0.9 ± 0.2	0.8 ± 0.3	0.7 ± 0.0	1.1 ± 0.1	1.1 ± 0.3	6.7 ± 4.6	0.7 ± 0.3
KC	3.5 ± 2.6	0.8 ± 0.2	0.9 ± 0.1	0.8 ± 0.2	1.0 ± 0.2	0.9 ± 0.1	1.3 ± 0.4	1.0 ± 0.2
IL-6	3.2 ± 2.3	***0.5 ± 0.1****	1.3 ± 0.3	0.9 ± 0.2	1.0 ± 0.1	1.2 ± 0.6	1.1 ± 0.5	1.2 ± 0.5
Cyp1a1	0.6 ± 0.2	***0.1 ± 0.1****	**76 ± 23***	**65 ± 33***	1.3 ± 0.5	1.8 ± 0.7	**368 ± 298***	**189 ± 152***

CBNP did not induce oxidative stress or a pro-inflammatory response in intrapulmonary airways.

Data show the n-fold increase of mRNA expression in the proximal and distal airways after CBNP exposure compared to medium controls. The concentration of 10 µg/ml CBNP was tested. Data are mean ± SEM. n=3-6, *p<0.05 CBNP compared to medium controls analyzed by Mann Whitney U test; *Gpx3*=Glutathione peroxidase 3, *Gr*=Glutathione reductase, *KC*=keratinocyte chemoattractant, *IL-6*=interleukin-6, *Cyp1a1*=Cytochrome P450 subtype 1a1
