# Supplementary material for: Biological effects of carbon black nanoparticles are changed by surface coating with polycyclic aromatic hydrocarbons
Source: Part Fibre Toxicol. 2017 Mar 21;14:8. doi: 10.1186/s12989-017-0189-1 (PMC5361723; doi:10.1186/s12989-017-0189-1)
Supplement: Supplementary file 14 — P90 agglomerates increased ciliary beat frequency and released mucus impaired particle transport speed. (PDF 573 kb) [file 12989_2017_189_MOESM12_ESM.pdf]

Additional file 12

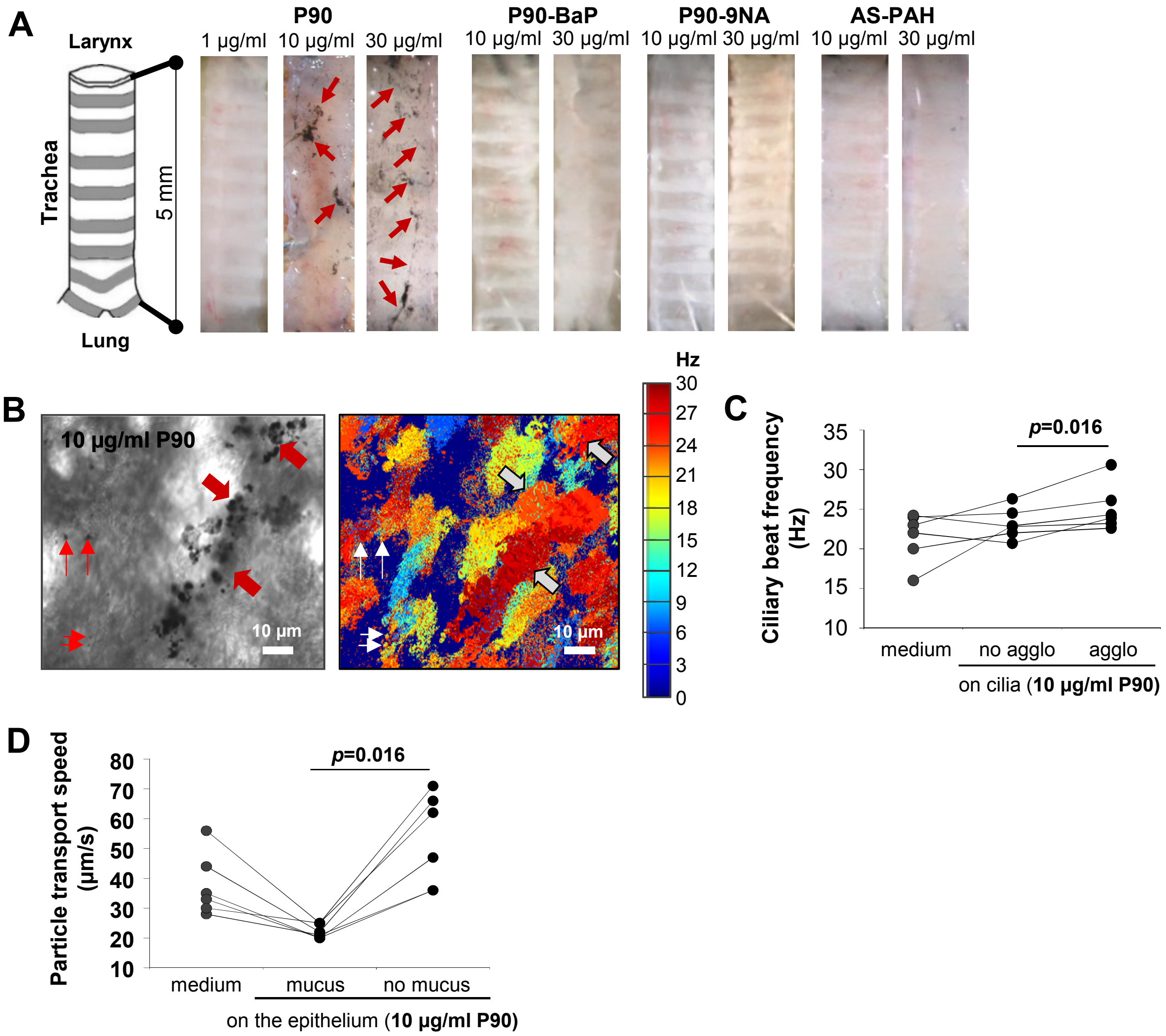

P90 agglomerates increased ciliary beat frequency and released mucus impaired particle transport speed.

A) Macroscopic visible CBNP agglomerates after exposure to 10 µg/ml and 30 µg/ml P90 (indicated by red arrows). No agglomerates were visible after exposure to 1 µg/ml P90, 10 µg/ml and 30 µg/ml P90-BaP, P90-9NA or AS-PAH. **B**) After exposure to 10 µg/ml P90, agglomerates attached to ciliated cells (red arrows). Color-coded images show the ciliary beat frequency of cilia with attached agglomerates (white arrows). **C**) Ciliary beat frequency after exposure to 10 µg/ml P90. Ciliary beat frequency of ciliated cells without and with microscopic visible agglomerates were measured. agglo=agglomerates **D**) Particle transport speed was analyzed in areas with and without P90 agglomerates after exposure to 10 µg/ml P90. Each point represents the mean particle transport speed of added polystyrene particles in the analyzed regions.

Exposure time was 24 hours. $p<0.05$ was considered statistically significant analyzed by Wilcoxon signed-rank test.
