# Supplementary material for: Biological effects of carbon black nanoparticles are changed by surface coating with polycyclic aromatic hydrocarbons
Source: Part Fibre Toxicol. 2017 Mar 21;14:8. doi: 10.1186/s12989-017-0189-1 (PMC5361723; doi:10.1186/s12989-017-0189-1)
Supplement: Supplementary file 15 — P90 did not cause agglomerates at 1 μg/ml, but released mucus impaired particle transport. (PDF 1144 kb) [file 12989_2017_189_MOESM15_ESM.pdf]

Additional file 15

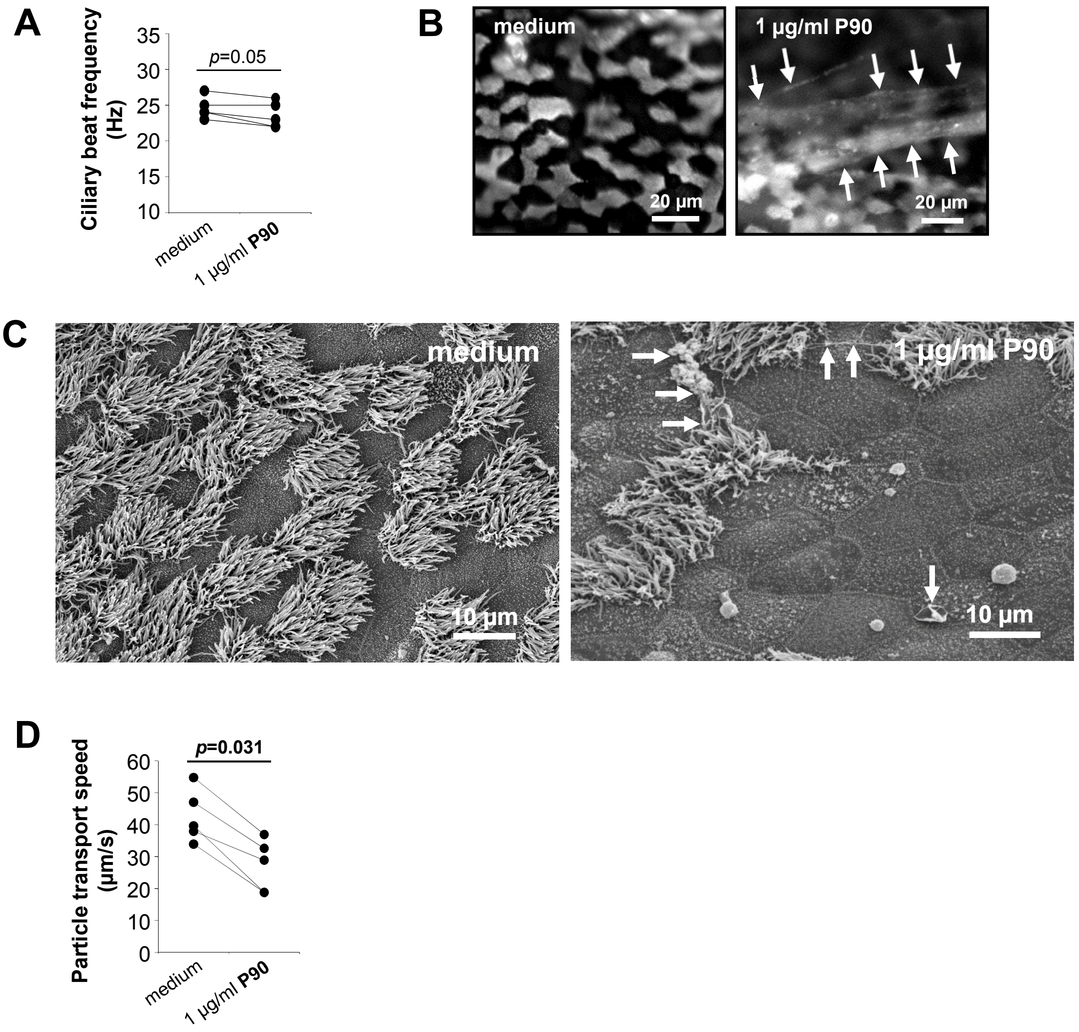

P90 did not cause agglomerates at 1 µg/ml, but induced mucus release that impaired particle transport.

A) Results of ciliary beat frequency analysis after incubation with 1 µg/ml P90. Each point represents the mean ciliary beat frequency of at least 50 ciliated cells measured at eight different tracheal regions of each animal. **B**) Representative images of stained mucus (WGA, UEA-1) after exposure to medium and 1 µg/ml P90. White arrows indicate mucus structures. **C**) Representative scanning electron microscopic images of tracheal epithelium after 24 hours exposure with medium and 1 µg/ml P90. White arrows indicate mucus structures. **D**) Results of particle transport speed analysis after exposure to 1 µg/ml P90 compared to medium. Each point represents the mean particle transport speed of added polystyrene particles in the analyzed regions.

Exposure time was 24 hours. $p<0.05$ was considered statistically significant analyzed by Wilcoxon signed-rank test.
