# Supplementary material for: Biological effects of carbon black nanoparticles are changed by surface coating with polycyclic aromatic hydrocarbons
Source: Part Fibre Toxicol. 2017 Mar 21;14:8. doi: 10.1186/s12989-017-0189-1 (PMC5361723; doi:10.1186/s12989-017-0189-1)
Supplement: Supplementary file 17 — P90-9NA induced cell death at the higher concentration. (PDF 641 kb) [file 12989_2017_189_MOESM17_ESM.pdf]

Additional file 17

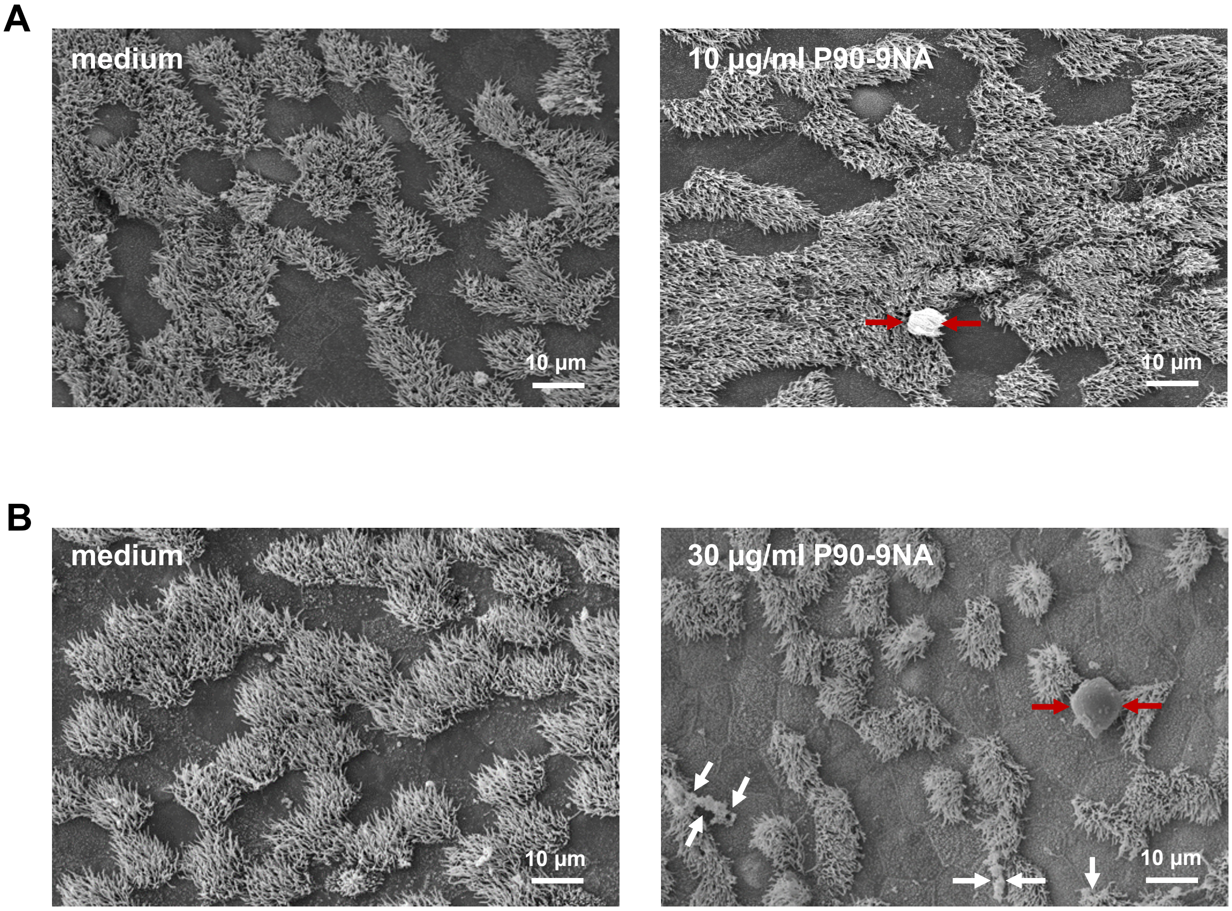

P90-9NA induced cell death at the higher concentration.

Representative images of scanning electron microscope analysis of tracheal epithelium after exposure to 10 µg/ml and 30 µg/ml P90-BaP compared to medium control. White arrows indicate mucus structures. Red arrows indicate dead cells.
