# Supplementary material for: Biological effects of carbon black nanoparticles are changed by surface coating with polycyclic aromatic hydrocarbons
Source: Part Fibre Toxicol. 2017 Mar 21;14:8. doi: 10.1186/s12989-017-0189-1 (PMC5361723; doi:10.1186/s12989-017-0189-1)
Supplement: Supplementary file 18 — P90 induced mucus release. (PDF 1021 kb) [file 12989_2017_189_MOESM18_ESM.pdf]

Additional file 18

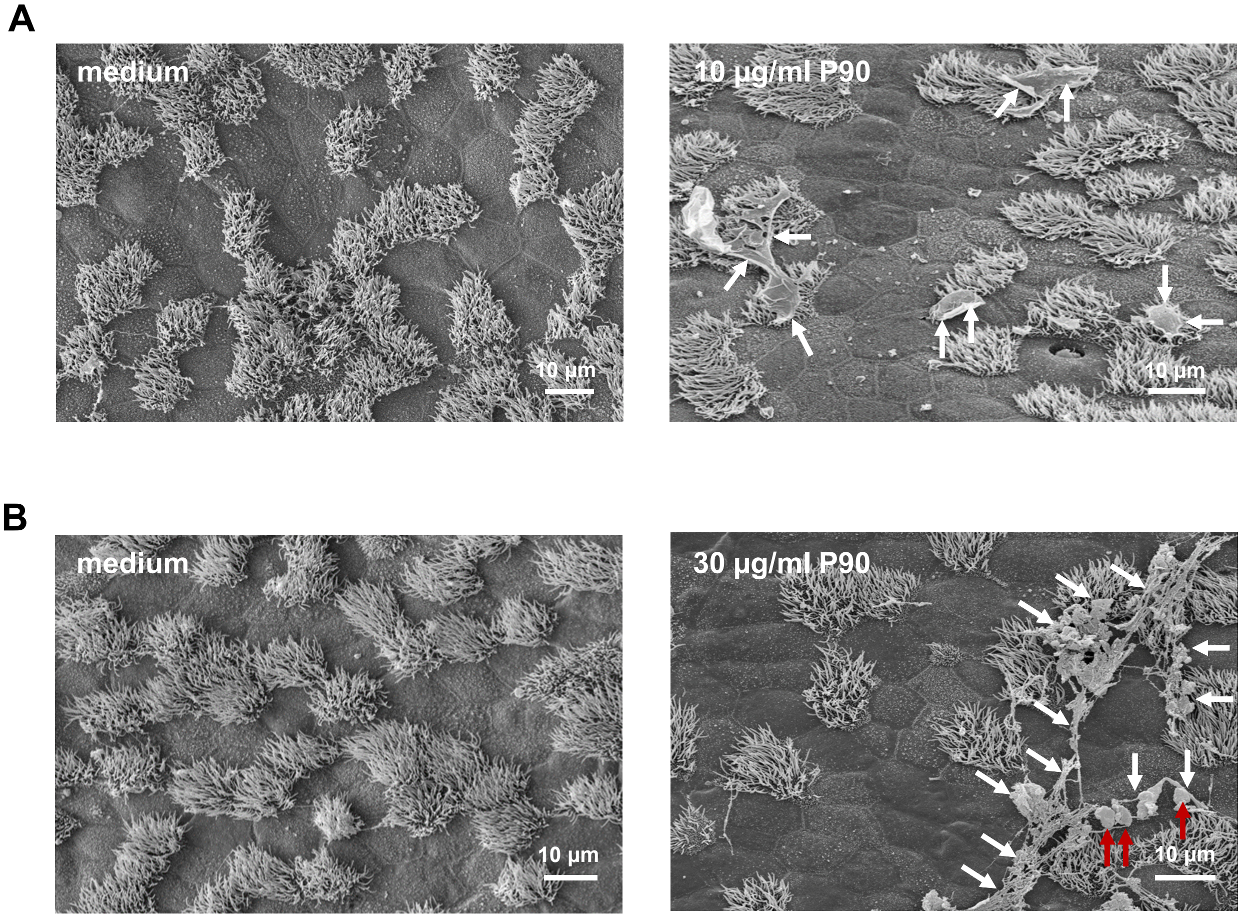

P90 induced mucus release.

Representative images of scanning electron microscope analysis of tracheal epithelium after exposure to 10 µg/ml and 30 µg/ml P90 compared to medium control. White arrows indicate mucus structures, red arrows indicate cell debris.
