# Supplementary material for: Biological effects of carbon black nanoparticles are changed by surface coating with polycyclic aromatic hydrocarbons
Source: Part Fibre Toxicol. 2017 Mar 21;14:8. doi: 10.1186/s12989-017-0189-1 (PMC5361723; doi:10.1186/s12989-017-0189-1)
Supplement: Supplementary file 19 — AS-PAH induced mucus release and cell death. (PDF 639 kb) [file 12989_2017_189_MOESM19_ESM.pdf]

Additional file 19

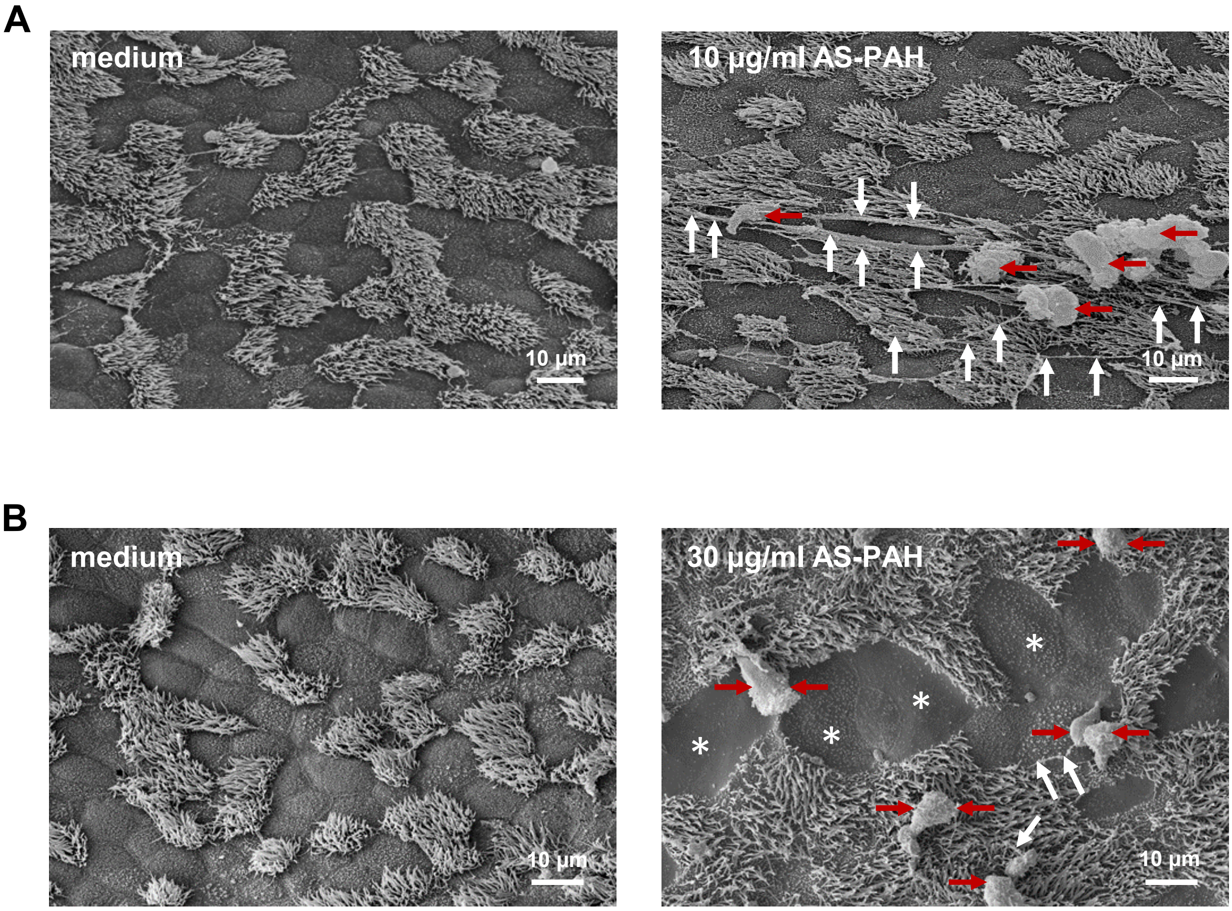

AS-PAH induced mucus release and cell death.

Representative images of scanning electron microscope analysis of tracheal epithelium after exposure to 10 µg/ml and 30 µg/ml AS-PAH compared to medium control. White arrows indicate mucus structures. Red arrows indicate dead cells. White asterisks indicate single elongated epithelial cells compensating epithelial cell damage.
