# Supplementary material for: Biological effects of carbon black nanoparticles are changed by surface coating with polycyclic aromatic hydrocarbons
Source: Part Fibre Toxicol. 2017 Mar 21;14:8. doi: 10.1186/s12989-017-0189-1 (PMC5361723; doi:10.1186/s12989-017-0189-1)
Supplement: Supplementary file 20 — No CBNP increased ciliary beat frequency at 30 μg/ml. (PDF 5594 kb) [file 12989_2017_189_MOESM20_ESM.pdf]

Additional file 20

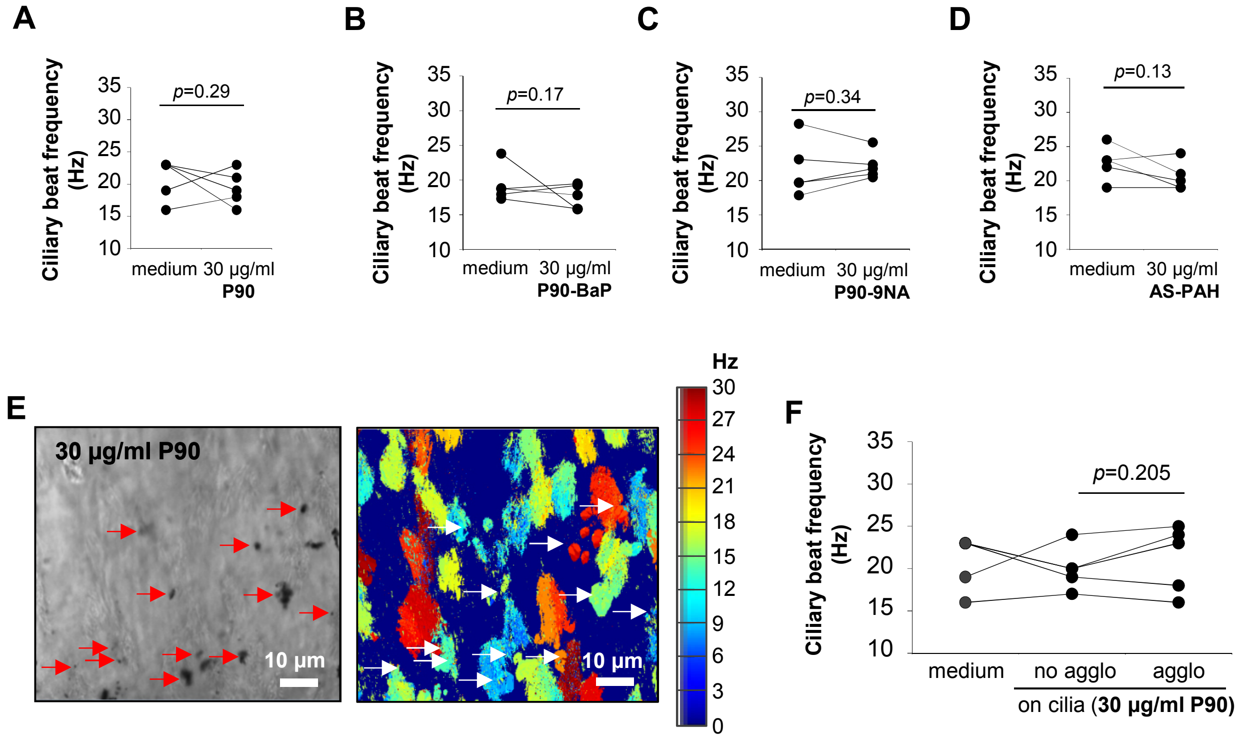

No CBNP induced ciliary beat frequency at 30 µg/ml.

A-D) Diagrams show the mean ciliary beat frequency after exposure to 30 µg/ml P90 (**A**), P90-BaP (**B**), P90-9NA (**C**) and AS-PAH (**D**) compared to medium controls. Each point represents the mean ciliary beat frequency of at least 50 ciliated cells measured at eight different tracheal regions of each animal. **E**) CBNP agglomerates attached to cilia of ciliated cells after exposure to 30 µg/ml P90 (left, red arrows). Color-coded image shows the ciliary beat frequency of ciliated cells with agglomerates (right, white arrows). **F**) Ciliary beat frequency of ciliated cells without and with microscopic visible agglomerates were analyzed after exposure to 30 µg/ml P90. agglo=agglomerates

Exposure time for all experiments was 24 hours. $p<0.05$ was considered statistically significant. The medium controls were compared to CBNP exposure analyzed by Wilcoxon signed-rank test.
